# Quantitative mapping of NRF-sMAF-ARE interactions reveals biphasic regulation of NRF2-dependent transcription by MAFG

**DOI:** 10.1016/j.redox.2026.104474

**Published:** 2026-11-05

**Authors:** Yongyi Wei, Lewis Alexander, Haofeng Ye, Xiaoyi Zhu, Qiu Li, Ke Wang, Zhaoyuan Sun, Dichun Huang, Hendrik Luesch, Aikseng Ooi, Eli Chapman, Donna Zhang

**Affiliations:** a Department of Molecular Medicine, The Herbert Wertheim UF Scripps Institute for Biomedical Innovation and Technology, Jupiter, FL, USA; b Center for Inflammation Science and Systems Medicine, The Herbert Wertheim UF Scripps Institute for Biomedical Innovation and Technology, Jupiter, FL, USA; c Department of Pharmacology and Therapeutics, University of Florida, Florida, USA; d Department of Pharmacology and Toxicology, College of Pharmacy, University of Arizona, Tucson, AZ, USA; e Department of Medicinal Chemistry and Center for Natural Products, Drug Discovery and Development (CNPD3), College of Pharmacy, University of Florida, Gainesville, FL, USA; f University of Florida Health Cancer Center, University of Florida, Gainesville, FL, USA; g Program in Cancer and Stem Cell Biology, Duke-NUS Medical School, Singapore; h Department of Medicine, Division of Hematology Oncology, College of Medicine, University of Florida, Florida, USA

## Abstract

Transcription factors regulate gene expression through coupled protein-protein and protein-DNA interactions, yet how these interactions collectively determine the formation of NRF-sMAF-ARE complexes that support transcription remains poorly understood. Here, we quantitatively resolved the nuclear factor erythroid 2-related factor (NRF)-small musculoaponeurotic fibrosarcoma (sMAF)-antioxidant response element (ARE) network using pair-resolved NanoBRET measurements of dimerization, protein-DNA association, and complex-formation kinetics. The detected dimer set consisted of NRF-sMAF heterodimers and sMAF-sMAF dimers, whereas NRF-NRF interactions remained at baseline across the tested concentration range. Within this detected dimer set, ARE recognition depended on the specific dimer-sequence combination, with core ARE substitutions altering apparent association, dissociation, and complex stability. Order-of-addition experiments further showed that NRF2-sMAF-ARE complexes could form from pre-existing sMAF-sMAF-ARE complexes, consistent with remodeling among competing DNA-bound complexes. An experimentally parameterized occupancy model predicted that NRF-sMAF-ARE occupancy is jointly controlled by NRF abundance, sMAF abundance, and ARE sequence and, specifically for NRF2 and MAFG, that increasing MAFG would first promote and then constrain NRF2-MAFG-ARE occupancy as the balance among competing MAFG-containing complexes shifted. Consistent with this prediction, cellular reporter measurements directly demonstrated a biphasic response to increasing MAFG expression inputs, with lower inputs enhancing and higher inputs reducing NRF2-dependent transcriptional activity. Together, these findings define a hierarchical mechanism of complex formation in which partner compatibility limits the dimer species available for ARE binding, DNA sequence and kinetics shape complex stability, dynamic remodeling connects competing DNA-bound complexes, and relative NRF-sMAF abundance determines NRF-sMAF-ARE occupancy, providing a general biochemical mechanism for coupled transcription-factor complex formation.

## 1 Introduction

Nuclear factor erythroid 2-related factor (NRF) family transcription factors coordinate cellular responses to oxidative, metabolic, and proteotoxic stress and regulate pathways involved in redox homeostasis, metabolism, protein quality control, and cell survival [1], [2], [3], [4]. Dysregulation of NRF signaling is strongly associated with human disease, including metabolic disorders and cancer [5], [6], [7], [8]. NRF1, NRF2, and NRF3 belong to the Cap ‘n’ Collar basic leucine zipper transcription-factor family and require sMAF proteins to form NRF-sMAF heterodimers that bind AREs and activate transcription [9], [10], [11]. The biological output of this system therefore depends not only on NRF abundance or activity, but also on the identity and availability of sMAF partners and the DNA sequences occupied by the resulting dimers [12], [13], [14].

The NRF-sMAF-ARE network contains competing dimers and DNA-bound complexes [15], [16], [17], [18]. NRF-sMAF heterodimers activate ARE-dependent transcription, whereas sMAF homodimers, which lack transcriptional activation domains, can occupy related ARE sequences and compete with transcriptionally activating NRF-sMAF-ARE complexes [19], [20]. How dimer identity, ARE sequence, and binding kinetics determine which dimers and DNA-bound complexes form, persist, and exchange remains incompletely understood [21], [22]. In this study, we use MAFG as the principal model for detailed binding, kinetic, and occupancy analyses because MAFG is an NRF2 target gene [23], [24], [25], which creates a potential feedback relationship in which NRF2-induced MAFG expression can both promote formation of NRF2-MAFG heterodimers that support ARE-dependent transcription and increase competing MAFG-containing dimers and DNA-bound complexes. However, the quantitative biochemical principles that govern changes in the relative occupancy of competing DNA-bound complexes and thereby tune downstream transcriptional responses have not been resolved.

More broadly, stress-responsive basic leucine zipper networks often change the relative occupancy of partner-dependent DNA-bound complexes in response to changes in transcription-factor abundance. Similar partner-dependent competition occurs during activating transcription factor 4-dependent induction of C/EBP homologous protein (CHOP/DDIT3) and activating transcription factor 3 (ATF3), as well as in activator protein 1 (AP-1)-associated stress responses [26], [27], [28], [29], [30]. However, most biochemical measurements examine dimerization, DNA binding, or kinetics separately and therefore do not reveal how these properties combine to determine which dimers and DNA-bound complexes predominate within coupled dimerization and DNA-binding networks [31], [32], [33]. Resolving the NRF-sMAF system requires measurements that distinguish specific dimerization and protein-DNA associations while preserving the competing equilibria among them.

Here, we used pair-resolved NanoBRET measurements [34], [35], [36], [37] to resolve the dimers and DNA-bound complexes underlying hierarchical control of NRF-sMAF-ARE complex formation by defining dimerization, DNA recognition, DNA-bound complex stability, and exchange between pre-existing DNA-bound complexes. We show that dimer identity determines which dimer species are available for ARE binding, ARE sequence differentially shapes the affinity and stability of alternative DNA-bound dimers, and order-of-addition experiments demonstrate that pre-existing sMAF-sMAF-ARE complexes can be converted through reversible exchange into NRF2-sMAF-ARE complexes. Integration of these measurements into an occupancy model further explains how the relative abundance of NRF and sMAF proteins can both promote and constrain NRF-sMAF-ARE occupancy. Consistent with the model prediction, cellular ARE-reporter assays demonstrated a biphasic response to increasing MAFG expression inputs, with lower inputs enhancing and higher inputs reducing NRF2-dependent reporter activity. Together, these findings establish a hierarchical mechanism showing how coupled protein-protein and protein-DNA interactions govern transcription-factor complex formation, exchange, and NRF-sMAF-ARE occupancy.

## 2 Results

### 2.1 Pair-resolved measurement of dimerization and protein-DNA association within the NRF-sMAF-ARE interaction network

The NRF-sMAF-ARE network contains multiple coexisting dimers and DNA-bound complexes that cannot be distinguished by bulk binding measurements alone (Fig. 1A). To resolve specific dimerization and DNA-association events within this network, we established a NanoLuc bioluminescence resonance energy transfer (NanoBRET) strategy based on proximity-dependent energy transfer between a NanoLuc donor and a fluorophore acceptor (Fig. 1B) [38].

**Fig. 1:**
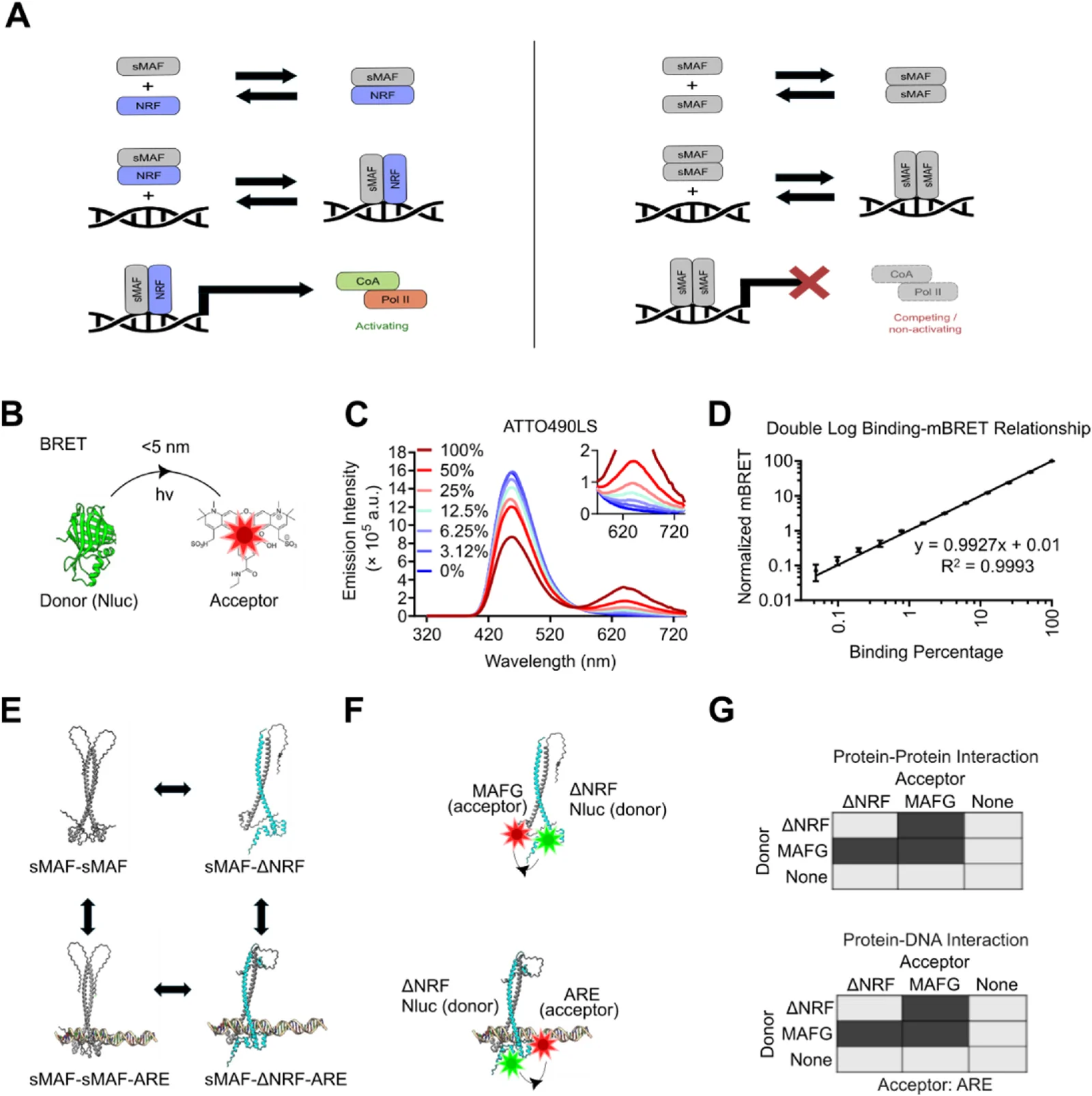
**NanoBRET platform for quantitative measurement of NRF-sMAF-ARE complex formation. (A)** Overview of the coupled NRF-sMAF-ARE equilibrium network. **(B)** NanoBRET detection principle based on proximity-dependent energy transfer between an Nluc donor and fluorophore acceptor. **(C)** ATTO490LS emission spectra across defined fractions of donor molecules paired with an acceptor. **(D)** Linear relationship between normalized mBRET and paired-donor fraction (R^2^ = 0.9993). **(E)** Protein-protein configuration: an Nluc-tagged NRF or sMAF donor and an NbALFA-fused partner labeled through ALFA-ATTO490LS report proximity within a dimer. **(F)** Protein-DNA configuration: an Nluc-tagged NRF or MAFG donor and ATTO590-labeled ARE report formation of NRF-MAFG-ARE or MAFG-MAFG-ARE complexes in the presence of unlabeled MAFG. **(G)** Binary map of the complete set of candidate NRF-sMAF molecular species with and without ARE. All logically possible dimeric and ARE-associated configurations are shown; dark markers indicate detectable NanoBRET signals, whereas configurations without dark markers were not detected under the corresponding assay conditions. Reciprocal NRF-NRF configurations were directly tested and remained at baseline up to 316 nM; their absence from the positive map reflects the measured baseline result rather than an untested configuration.

We first evaluated whether the NanoBRET signal quantitatively reflected the fraction of donor molecules paired with an acceptor. Across defined fractions of donor molecules paired with an acceptor, ATTO490LS produced the expected decrease in donor emission and increase in acceptor emission (Fig. 1C; Fig. S1; Table S1). Normalized milliBRET (mBRET) increased linearly with the paired-donor fraction (R^2^ = 0.9993), supporting quantitative conversion of the NanoBRET readout into the fraction of donor molecules paired with an acceptor (Fig. 1D).

Additional analyses showed that the ratiometric response remained robust across 10–200 μL in 96-well plates and 10–50 μL in 384-well plates, and across the tested donor concentrations and common buffer components (Fig. S2). Across the 20-fold volume range in 96-well plates, the 620/420 ratio changed by only approximately 10% relative to the 100 μL condition, normalized 50% pairing samples varied by less than 2%, and transfer to 384-well plates produced comparable ratios. The ratiometric readout also remained constant across a 3000-fold donor dilution and a 1000-fold acceptor range above the NbALFA-ALFA dissociation constant. The defined 0%, 50%, and 100% acceptor-labeled fractions remained distinguishable. At low acceptor concentrations, the signal decreased as NbALFA–ALFA binding became incomplete, showing that the readout also responds to the underlying binding equilibrium. At a 1% donor–acceptor pairing fraction, the Z′ factor exceeded 0.9, indicating robust separation between the paired and unpaired controls. Together, these results established a quantitative platform for resolving specific dimerization and protein-DNA association events within the NRF-sMAF-ARE interaction network [39], [40].

We then used two complementary donor–acceptor configurations to resolve the full set of candidate molecular species generated by the coupled NRF-sMAF-ARE network. Because NRF and sMAF proteins can form alternative dimers, and each dimeric combination can occur either without DNA or in association with ARE, the network contains a large combinatorial set of protein-only and DNA-containing species that coexist in the same reaction and are difficult to distinguish using conventional bulk binding measurements. For protein-protein measurements, an Nluc-tagged NRF or sMAF donor was paired with an NbALFA-fused partner bound to ALFA-ATTO490LS; this configuration assigned the NanoBRET signal to a defined dimeric pair (Fig. 1E). For protein-DNA measurements, an Nluc-tagged NRF or MAFG donor was paired with an ATTO590-labeled ARE; in the presence or absence of the relevant unlabeled partner, this configuration distinguished ARE-associated species according to their protein composition (Fig. 1F). Together, the two readout geometries covered the logically possible dimeric and ARE-containing configurations in the network and enabled them to be evaluated individually rather than as an unresolved ensemble. Fig. 1G organizes this complete candidate-species space and overlays the qualitative detection outcome for each configuration examined: NRF-sMAF and sMAF-sMAF dimers and their ARE-bound complexes produced detectable NanoBRET signals, whereas NRF-NRF pairs and the tested monomer-associated protein-DNA configurations remained at baseline. Baseline configurations were therefore classified as not detected under the measured assay conditions, without implying that the corresponding molecular species are physically impossible under all conditions.

### 2.2 NRF-sMAF dimerization defines the dimers available for ARE binding

We first defined which transcription-factor dimers are available to enter the NRF-sMAF-ARE interaction network. Sequence alignment of NRF1-3 and the sMAF family revealed conserved basic-region and leucine-zipper residues known to mediate NRF-sMAF heterodimerization and sMAF homodimerization (Fig. 2A) [41]. To measure these interactions without directly modifying the leucine-zipper interface, ΔNRF fragments containing the Neh1/Neh3 regions and full-length sMAFs were fused to reciprocal Nluc donor and NbALFA acceptor fusions, with ALFA-ATTO490LS bound to NbALFA (Fig. 2B) [42]. With donor fixed at 10 pM and the NbALFA-fused partner titrated from 0 to 316 nM, this design measured dimerization in both donor-acceptor orientations (Fig. 2C).

**Fig. 2:**
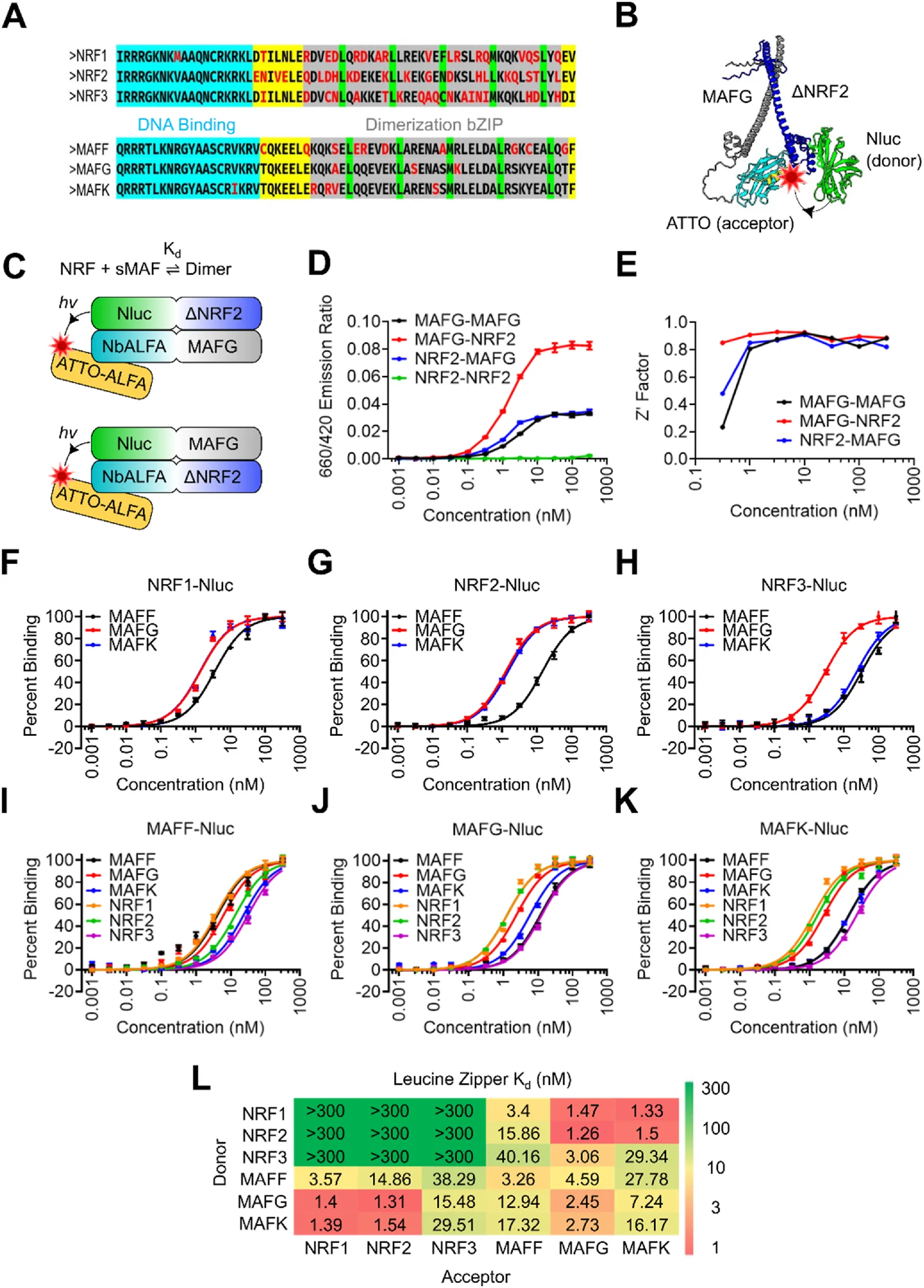
**NanoBRET mapping of NRF-sMAF dimerization. (A)** Alignment of human NRF and sMAF basic regions and leucine zipper domains. **(B, C)** Reciprocal protein-protein NanoBRET configurations using Nluc donor fusions and NbALFA-fused partners labeled with ALFA-ATTO490LS. **(D, E)** Representative NRF2-MAFG binding modes and assay-performance analysis. **(F-K)** NbALFA-fused partner titrations (0–316 nM) against 10 pM Nluc-tagged NRF1, NRF2, NRF3, MAFF, MAFG, and MAFK donors. **(L)** Heatmap of apparent *K*_d_ values from single-site binding fits across the full donor-acceptor matrix. Data represent mean ± SD from triplicate independent experiments; error bars are not visible where they fall within the symbol size. Baseline-level signals indicate no detectable donor-acceptor NanoBRET signal under these conditions, including all tested NRF-NRF pairs.

Analysis of the NRF2-MAFG subset revealed saturable NRF2-MAFG, MAFG-NRF2, and MAFG-MAFG interactions, whereas NRF2-NRF2 remained at baseline up to 316 nM (Fig. 2D; Fig. S3). For the detectable interactions, Z′ exceeded 0.8 at titrated-partner concentrations above 1 nM, demonstrating strong separation from the corresponding baseline controls (Fig. 2E). We then titrated the six acceptor partners against each Nluc-tagged NRF1, NRF2, NRF3, MAFF, MAFG, and MAFK donor (Fig. 2F–K; Table S2). Across the full matrix, NRF proteins formed detectable complexes with sMAF partners but not with other NRF proteins, whereas sMAF proteins formed both NRF-sMAF heterodimers and sMAF-sMAF dimers.

The resulting apparent dissociation-constant matrix revealed a clear interaction hierarchy (Fig. 2L). NRF1 and NRF2 formed the strongest heterodimers with MAFG and MAFK; for example, the apparent *K*_d_ values were 1.26 nM for NRF2–MAFG and 1.33 nM for NRF1–MAFK. NRF3 generally formed weaker sMAF heterodimers, exemplified by an apparent *K*_d_ of 29.34 nM for NRF3–MAFK. Among the sMAF homodimers, the apparent *K*_d_ values were 2.45 nM for MAFG–MAFG, 3.26 nM for MAFF–MAFF, and 16.17 nM for MAFK–MAFK. In contrast, all NRF–NRF combinations remained at baseline at concentrations up to 316 nM. These findings show that partner compatibility represents the first step in hierarchical complex formation by limiting the dimer species available for subsequent ARE binding.

### 2.3 Dimer identity and ARE sequence jointly determine DNA recognition

To determine how dimer identity and ARE sequence govern DNA recognition, we developed a protein-DNA NanoBRET assay in which NanoLuc-tagged ΔNRF fragments or full-length MAFG were paired with an ATTO590-labeled NQO1 ARE duplex (Fig. 3A and B) [43], [44]. Unlabeled MAFG (3 nM) was included with 10 pM Nluc-tagged donor to favor formation of the corresponding NRF-MAFG or MAFG-MAFG dimers before DNA binding. This design assigned the NanoBRET signal specifically to protein-DNA association while preserving the coupled dimerization equilibrium. WT NQO1 ARE produced saturable binding curves with apparent *K*_d_ values of 0.0762 nM for NRF2–MAFG–ARE and 1.96 nM for MAFG–MAFG–ARE (Fig. 3C). Because NanoBRET responses are sensitive to donor–acceptor geometry, we additionally evaluated full-length NRF2 using a reverse-labeling configuration in which Nluc was fused to MAFG and full-length NRF2 remained unlabeled. In this configuration, full-length NRF2 combined with His-tagged Nluc-MAFG or tag-cleaved Nluc-MAFG produced apparent *K*_d_ values of 0.0752 nM and 0.0801 nM, respectively (Fig. S4A). These values differed by only approximately 1.3% and 5.1%, respectively, from the apparent *K*_d_ of 0.0762 nM obtained with the primary Neh1-containing ΔNRF2–MAFG configuration. Thus, full-length NRF2 reproduced the ARE-binding behavior measured with the NRF2 DNA-binding module, and removal of the His tag from Nluc-MAFG did not appreciably alter ARE engagement under these assay conditions. Assay-performance analysis confirmed sufficient dynamic range and robustness for sequence-dependent comparisons (Fig. 3D).

**Fig. 3:**
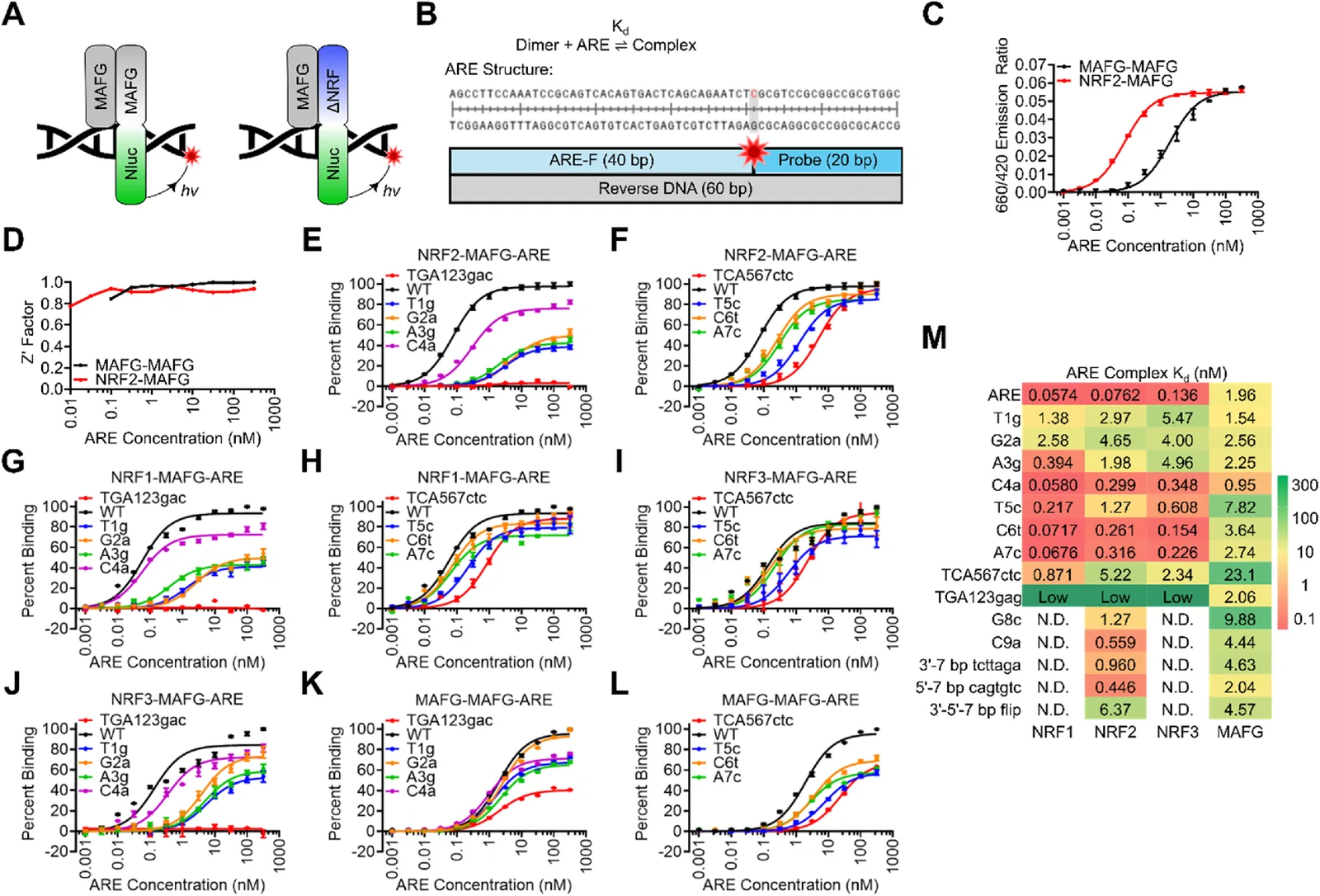
**Thermodynamic dissection of NRF-sMAF-ARE and sMAF-sMAF-ARE recognition. (A)** NanoBRET assay design for measuring ARE binding by NRF-sMAF and sMAF-sMAF complexes. ΔNRF fragments containing Neh1/Neh3 or full-length MAFG were N-terminally fused to NanoLuc and paired with 3 nM unlabeled MAFG to favor the relevant MAFG-containing dimer context before DNA binding**. (B)** Preparation of the ATTO590-labeled NQO1 ARE duplex probe. **(C, D)** Representative ARE-binding titrations and assay-performance analysis for NRF2-MAFG-ARE and MAFG-MAFG-ARE complexes. **(E–L)** Binding profiles for NRF2, NRF1, NRF3, and MAFG across ARE mutant groups, measured in the presence of unlabeled MAFG and normalized to WT ARE. **(M)** Heatmap of apparent dissociation constants *K*_d_ derived from single-site binding fits, comparing base-wise sequence sensitivity across NRF-MAFG heterocomplexes and MAFG-MAFG homocomplexes. Data represent mean ± SD from triplicate independent experiments; error bars are not visible where they fall within the symbol size, and baseline-level signals indicate no detectable binding.

We then profiled ARE binding by NRF2-MAFG, NRF1-MAFG, NRF3-MAFG, and MAFG-MAFG across the mutant panel and normalized each response to WT ARE (Fig. 3E–L; Fig. S4; Table S3). The resulting apparent *K*_d_ heatmap showed that ARE sequence changes were not uniformly tolerated across complexes (Fig. 3M). For example, the TCA567 ctc substitution increased the apparent *K*_d_ to 5.22 nM for NRF2-MAFG-ARE and 23.1 nM for MAFG-MAFG-ARE, approximately 68.5-fold and 11.8-fold increases, respectively. Thus, the substitution weakened apparent affinity in both dimer contexts, but by different magnitudes, demonstrating that sequence sensitivity depends on the dimer-ARE combination. Together, these measurements identify ARE sequence recognition as the second step in hierarchical complex formation, determining NRF-MAFG-ARE occupancy among the detected dimer species.

### 2.4 ARE substitutions alter apparent association and DNA-bound complex stability

Next, we extended the NanoBRET platform from equilibrium measurements to real-time kinetic analysis of NRF2-MAFG-ARE formation. NanoLuc-tagged NRF2 was preassembled with unlabeled MAFG, and association was initiated by injection of ATTO590-labeled ARE, allowing ternary-complex formation to be monitored immediately after DNA addition (Fig. 4A). To maximize temporal resolution, the rapid-acquisition condition was sampled at 0.1 s intervals, and kinetic traces were normalized against negative and pre-equilibrated reference conditions. This design allowed the early binding phase to be resolved without requiring simultaneous donor channel acquisition. WT ARE association traces were better described by a bi-exponential association function, resolving a rapid initial component and a slower secondary component (Fig. 4B, Fig. S5 and Table S4A). The direct observation of two kinetically distinguishable components indicates that NRF2-MAFG-ARE formation cannot be adequately explained by a simple one-step DNA-capture model. Because NanoBRET monitors NRF2–ARE proximity within a coupled protein–protein and protein–DNA assembly network, bi-exponential fitting provides a mathematical separation of kinetic components rather than a physical separation of molecular species or microscopic reactions. Based on the ARE-concentration dependence of the rapid component, we interpreted the fast phase as being dominated by DNA engagement and carried k_obs, fast_ forward for quantitative analysis of apparent ARE-association kinetics. The slower component was interpreted as a candidate protein-partner reorganization or exchange process within the coupled assembly network. These assignments represent a working mechanistic interpretation and do not imply that the fast and slow components contain exclusively protein–DNA and protein–protein contributions, respectively. Plotting k_obs, fast_ as a function of ARE concentration yielded an independent estimate of apparent k_on_, and this concentration-dependent approach agreed closely with values calculated from independently measured apparent *K*_d_ and fixed-concentration k_obs, fast_ measurements (Fig. 4C and D). The concentration-series and fixed-concentration approaches yielded apparent k_on_ values of 0.534 ± 0.097 and 0.563 ± 0.024 nM^−1^ s^−1^, respectively, a 6% difference with overlapping 95% confidence intervals. This agreement supports the interpretation that the fast component is dominated by ARE engagement without requiring it to represent a uniquely isolated microscopic DNA-binding step.

**Fig. 4:**
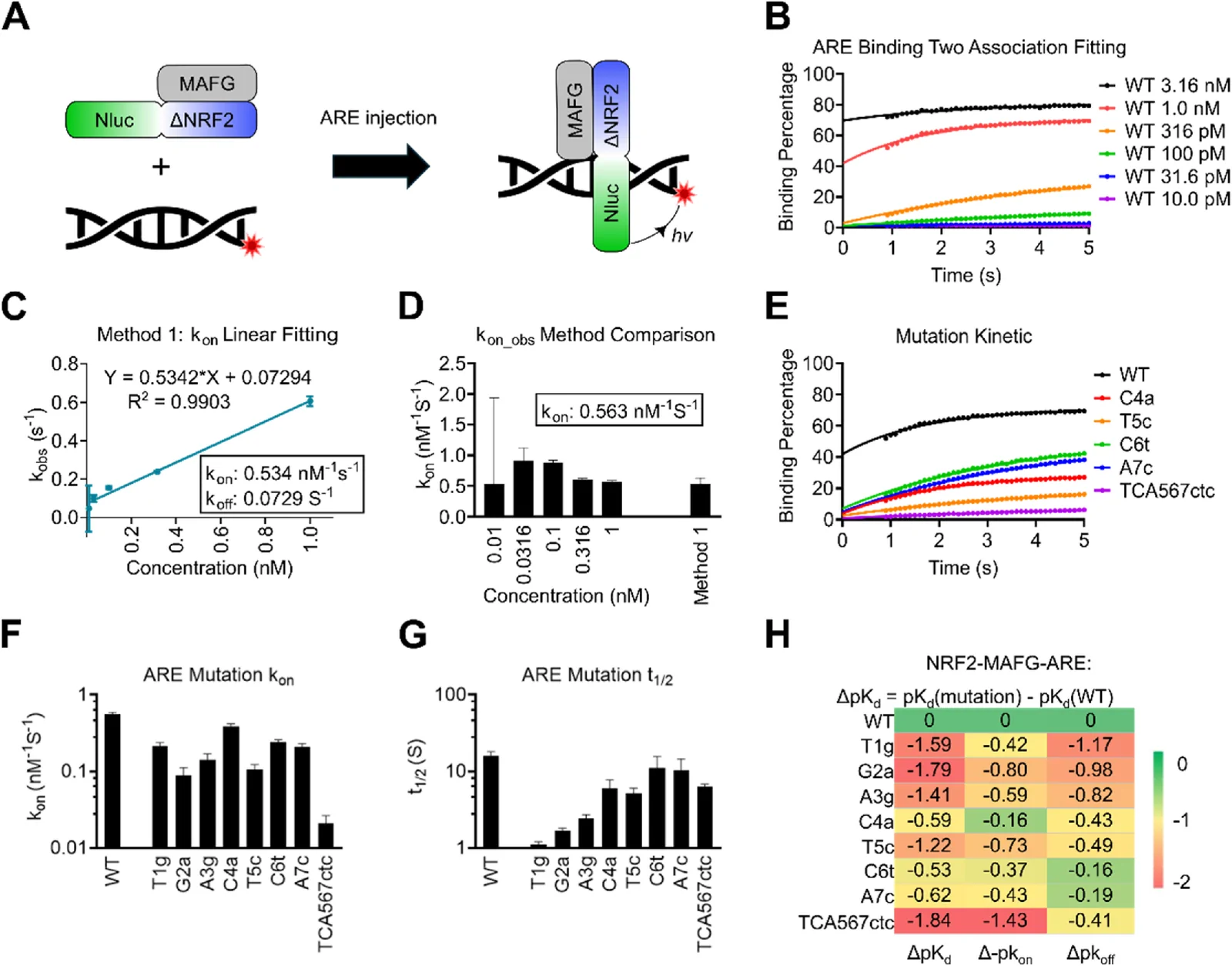
**Kinetic determinants of NRF-sMAF-ARE binding and sequence-dependent residence time. (A)** Schematic of the real-time NanoBRET association assay. NanoLuc-tagged NRF2 was preassembled with unlabeled MAFG, and binding was initiated by injection of ATTO590-labeled ARE to monitor NRF2-MAFG-ARE ternary complex formation. **(B)** Expanded early association phase of the WT ARE concentration series used for kinetic fitting. **(C)** Estimation of apparent association parameters from the concentration dependence of the observed association rate. **(D)** Comparison of apparent k_on_ values obtained from independent fitting approaches. **(E)** Representative association kinetics of WT and mutant ARE. **(F)** Sequence-dependent effects on apparent k_on_. **(G)** Sequence-dependent apparent residence half-life t_1/2_ = ln(2)/k_off_. Apparent k_off_ was calculated from apparent *K*_d_ and k_on_ rather than measured in a direct dissociation assay. **(H)** Integrated heatmap of thermodynamic and kinetic parameter shifts for ARE variants relative to the WT NQO1 ARE. Raw kinetic data are shown as points and fitted curves as solid lines. Summary data represent mean ± SD from independent experiments; error bars are not visible where they fall within the symbol size.

We then asked how ARE sequence changes affect the kinetic parameters of NRF2-MAFG-ARE formation. Association kinetics were measured across the ARE mutant panel, with representative WT and mutant traces shown in Fig. 4E. The same kinetic fitting workflow established for the WT analysis was applied to the mutant traces, and k_obs, fast_ was extracted from the selected fitting approach when the rapid kinetic component could be reliably constrained (Fig. S6 and Table S4B). The fitted k_obs, fast_ values were then combined with independently measured apparent *K*_d_ values to derive apparent k_on_ and k_off_ for mutant comparisons (Table S5). Here, apparent k_off_ was calculated from apparent *K*_d_ and apparent k_on_ according to k_off_ = *K*_d_ × k_on_; it is therefore a mathematically derived, condition-specific kinetic parameter rather than a directly recorded dissociation trace. For WT ARE, the fixed-concentration method yielded an apparent k_on_ of 0.563 nM^−1^ s^−1^ and a derived apparent k_off_ of 0.0429 s^−1^. Across single-base variants, apparent k_on_ ranged from 0.0889 to 0.389 nM^−1^ s^−1^, corresponding to approximately 1.45- to 6.33-fold lower values than WT. The multi-base TCA567 ctc variant had the lowest apparent k_on_, 0.0210 nM^−1^ s^−1^, approximately 26.8-fold lower than WT (Fig. 4F). Apparent k_off_ increased as high as 0.630 s^−1^, approximately 14.7-fold above WT. The corresponding apparent residence half-life, calculated as ln(2)/k_off_, decreased from approximately 16.2 s for WT to 1.10 s at this highest k_off_. Several core substitutions, particularly at positions 1–3, therefore strongly reduced DNA-bound complex stability (Fig. 4G). Integrated thermodynamic and kinetic parameter shifts further separated association-related effects from residence-time effects across WT and mutant AREs (Fig. 4H). Together, these results identify DNA-bound complex stability as the third determinant in hierarchical complex formation. Sequence substitutions altered both apparent association and derived dissociation parameters, and the larger changes in derived apparent k_off_ for several core substitutions support a prominent contribution of DNA-bound complex stability to ARE sequence discrimination.

### 2.5 Reversible exchange between ARE-bound complexes during formation of NRF-sMAF-ARE complexes

The biphasic kinetics raised the question of whether the slower component depended on how the coupled NRF2-MAFG-ARE system assembled. To test this, we changed the preincubation and order of component addition while maintaining the same final NRF2, MAFG, and ARE composition and monitoring the same NRF2–ARE NanoBRET readout. In one route, NRF2 was added to a pre-equilibrated MAFG/ARE mixture; in the other, MAFG was added to a pre-equilibrated NRF2/ARE mixture (Fig. 5A–C and Fig. S7). Each preincubated sample contained the equilibrium distribution generated by its components rather than a single isolated complex. Despite the identical final composition and readout, the apparent association kinetics differed between the two routes. MAFG injection gave an apparent association rate of approximately 500 nM^−1^ s^−1^, about twice the approximately 260 nM^−1^ s^−1^ measured after NRF2 injection (Fig. 5C). Because these values were calculated using the binary NRF2–MAFG interaction, they provide an operational protein-partner assembly/exchange scale without implying that protein–DNA processes were absent from either reaction. The dependence on assembly history is consistent with the working interpretation that the slower kinetic component is dominated by protein-partner reorganization or exchange rather than DNA capture alone.

**Fig. 5:**
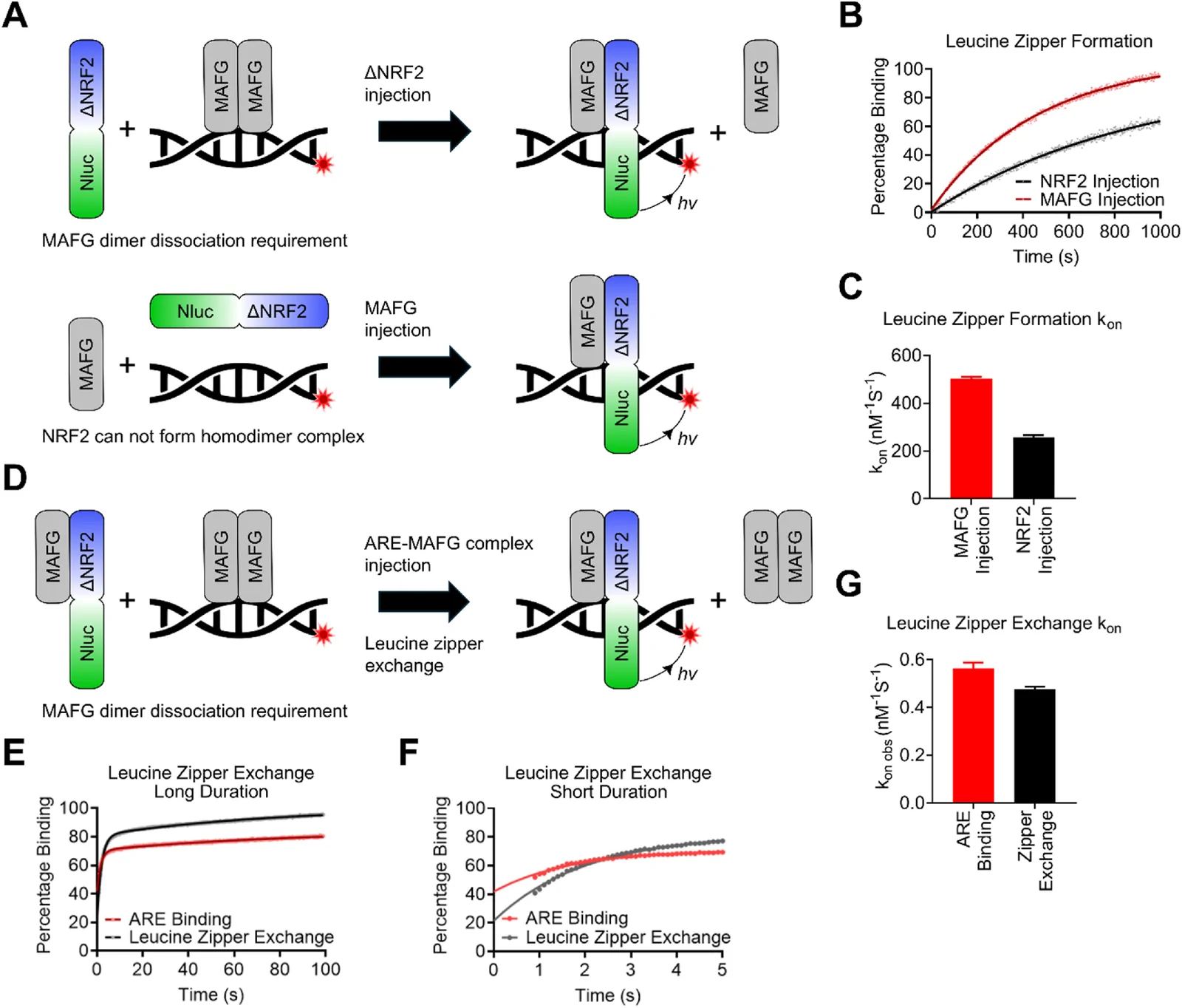
Assembly-history dependence and reversible remodeling during NRF-sMAF-ARE complex formation. **(A)** Alternative-order assay: NRF2 was added to a pre-equilibrated MAFG/ARE mixture, or MAFG to a pre-equilibrated NRF2/ARE mixture, with final composition and NRF2–ARE readout held constant. **(B)** Representative kinetics for the two routes. **(C)** Binary NRF2–MAFG-based apparent parameters reported on an operational protein-partner assembly/exchange scale. **(D)** Pre-occupied ARE assay combining separately pre-equilibrated NRF2/MAFG and MAFG/ARE mixtures. **(E)** Representative NRF2–ARE NanoBRET kinetics after mixing. **(F)** Comparison of NRF2-MAFG-ARE formation under pre-occupied and direct ARE-binding conditions. **(G)** ARE-derived apparent parameters reported on an operational protein–DNA kinetic scale. Both assays contain coupled protein–protein and protein–DNA reactions; neither parameter set represents isolated microscopic rate constants. Data are mean ± SD from independent experiments; error bars may fall within the symbols. Raw data are points and fitted curves are solid lines.

We next asked whether NRF2-MAFG-ARE could form when ARE had already equilibrated with MAFG. We preincubated an NRF2/MAFG mixture and a MAFG/ARE mixture separately and then combined them (Fig. 5D). Each mixture contained the equilibrium distribution generated by its components; the experiment did not require isolation or quantitative assignment of a single NRF2-MAFG or MAFG-MAFG-ARE species before mixing. The NRF2–ARE NanoBRET signal increased after the mixtures were combined, showing efficient formation of NRF2-MAFG-ARE under the pre-occupied ARE condition (Fig. 5E and F). The apparent rate was approximately 0.48 nM^−1^ s^−1^, compared with approximately 0.58 nM^−1^ s^−1^ for the direct ARE-binding route, a difference of roughly 17% (Fig. 5G). Because these values were calculated from ARE engagement, they define an operational protein–DNA kinetic scale without excluding simultaneous protein-partner reorganization. Their approximately three-order-of-magnitude difference from the values in Fig. 5C reflects the different effective constraints used in the protein–protein and protein–DNA calculations, rather than physical isolation of separate microscopic reactions. Together, the two experiments are consistent with a model in which the mathematically resolved fast component is dominated by DNA engagement, whereas the slow component is dominated by protein-partner reorganization or exchange. They also support reversible remodeling between pre-existing ARE-bound complexes while leaving the exact microscopic exchange route unresolved. Thus, NRF2-MAFG-ARE formation under the pre-occupied ARE condition is consistent with a branched complex-formation model, although whether exchange occurs without complete dissociation into free components remains unresolved.

### 2.6 Relative NRF and sMAF abundance determines sequence-dependent ARE occupancy

The experimentally measured dimerization and protein-DNA association parameters described above served as inputs to an experimentally parameterized occupancy calculation (Fig. 6A), which integrates dimerization, DNA binding, and competition to predict NRF-sMAF-ARE occupancy. Because these parameters were obtained under distinct assay compositions and readout configurations, they are condition-dependent descriptors of the measured NanoBRET readouts rather than elementary microscopic constants. We therefore used this experimentally parameterized model to calculate whether the measured interaction hierarchy was sufficient to generate concentration- and sequence-dependent occupancy responses.

**Fig. 6:**
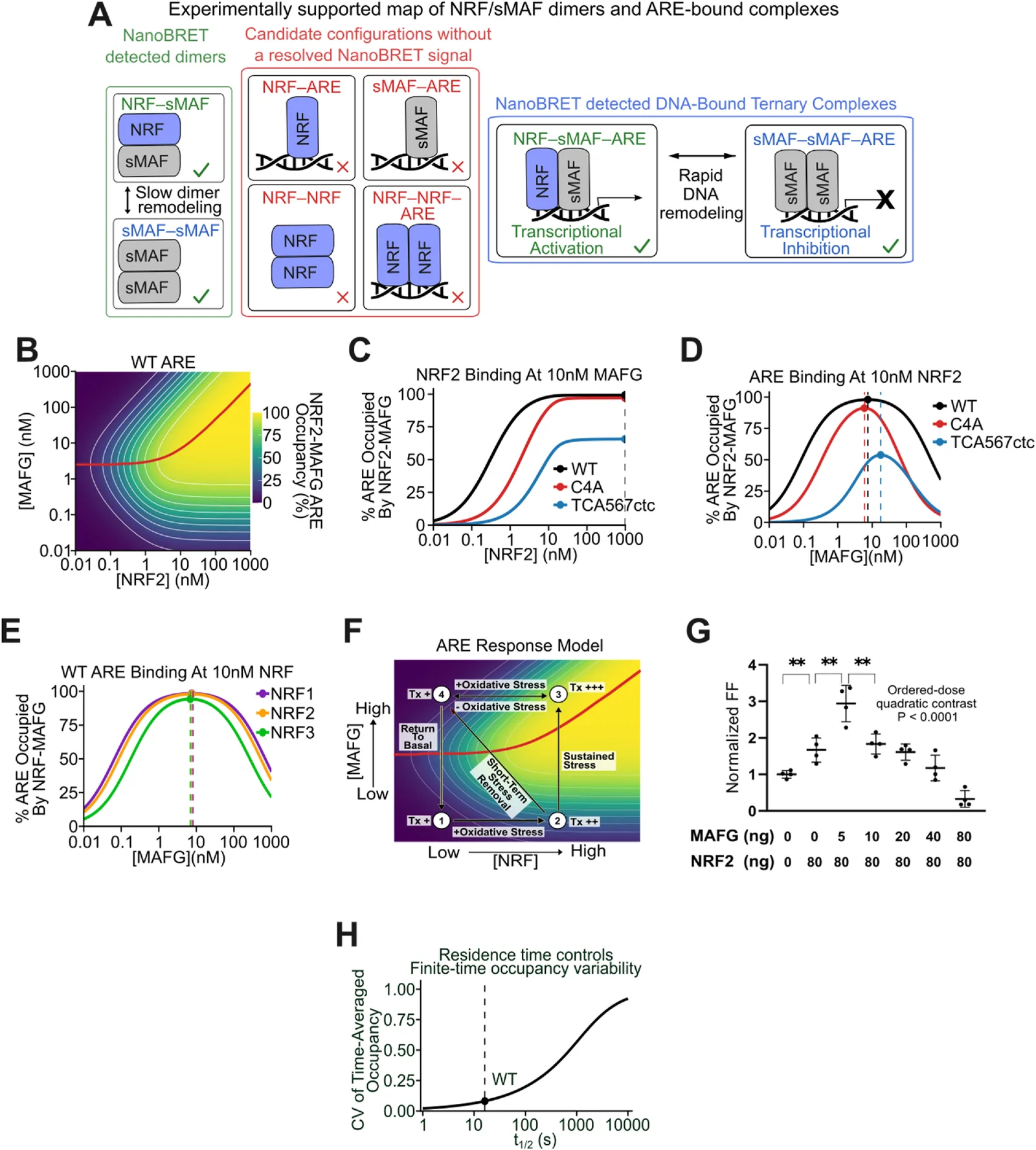
**Integrated occupancy model and cellular test of NRF2-MAFG-ARE regulation. (A)** Experimentally supported map of NRF-sMAF dimers, sMAF-sMAF dimers, and their ARE-bound complexes. NanoBRET-detected NRF-sMAF and sMAF-sMAF dimers define the dimer species considered in the occupancy model. NRF-NRF dimerization was not detected under the measured conditions, and no distinct monomer-associated protein-DNA association signal or monomer-associated kinetic phase was resolved. These species were therefore not included in the simplified occupancy model. **(B)** Calculated, model-predicted WT ARE occupancy surface for NRF2-MAFG-ARE as a function of NRF2 and MAFG concentrations. **(C, D)** Calculated occupancy responses for WT, C4A and TCA567 ctc ARE sequences when NRF2 or MAFG is varied while the other component is held constant. **(E)** Calculated, model-predicted occupancy responses for WT ARE with NRF1, NRF2, or NRF3 while MAFG concentration was varied. **(F)** Model-defined WT NRF2-MAFG-ARE concentration regimes illustrating predicted transitions among basal-like regime 1, acute-activation-like regime 2, sustained-stress regime 3, and recovery-like regime 4. **(G)** Effect of MAFG expression on NRF2-dependent ARE reporter activity. HEK-293 cells were transiently transfected with 20 ng of a firefly luciferase reporter containing the ARE sequence CCTGAGTCACG, 80 ng of pCI-NRF2-K7, and increasing amounts of pCI-MAFG (0–80 ng). Empty pCI vector was added as needed to maintain a constant total of 220 ng plasmid DNA per well across all conditions. The empty-vector control contained the ARE reporter and empty pCI vector without NRF2-K7 or MAFG. Firefly luciferase activity was measured 16 h after transfection and normalized to the empty-vector control. Data are presented as mean ± SD from four independently transfected wells in one representative experiment, with individual values shown. The experiment was independently repeated three times with similar results. The ordered-dose quadratic contrast was significant (P < 0.0001); pairwise comparisons indicated by brackets were assessed using two-tailed unpaired Student's t tests (**P < 0.01). **(H)** Illustrative kinetic sampling model showing how derived residence-time effects can be related to finite-time occupancy variability. Equilibrium occupancy was fixed at pocc = 0.5 while residence half-life was varied, and the coefficient of variation of time-averaged occupancy was calculated over a 3600-s observation window. The derived apparent residence half-life of WT NRF2-MAFG-ARE is indicated as an experimental reference point.

Using the experimentally derived apparent parameters for the WT NRF2-MAFG-ARE system, the model generated a predicted occupancy surface across relative NRF2 and MAFG inputs (Fig. 6B). Within this model, NRF2-MAFG-ARE occupancy depended on the relative balance between NRF2 and MAFG rather than on NRF2 alone. When MAFG was held constant, increasing NRF2 produced a monotonic increase in NRF2-MAFG-ARE occupancy (Fig. 6C). In contrast, at each fixed NRF2 input, NRF2-MAFG-ARE occupancy increased and then declined as MAFG input increased, producing a single maximum (Fig. 6D). This biphasic response arises because MAFG is required to form NRF2-MAFG heterodimers that occupy the ARE, whereas increasing MAFG also promotes competing MAFG-containing complexes that reduce NRF2-MAFG-ARE occupancy. These concentration-response curves are computational outputs of the experimentally parameterized model rather than direct occupancy measurements.

ARE sequence further altered this concentration response. Compared with WT NQO1 ARE, the TCA567 ctc mutant, representing a TGACXXXGC-type ARE variant [45], [46], [47], [48], produced lower predicted occupancy in the lower-NRF2 region when MAFG was held constant (Fig. 6C). At fixed NRF2 input, the occupancy maximum occurred at a higher relative MAFG input for TCA567 ctc than for WT NQO1 ARE, thereby extending the model input range over which NRF2-MAFG-ARE occupancy remained elevated (Fig. 6D). Additional comparisons are shown in Fig. S8. Thus, ARE sequence does not simply scale binding strength up or down but alters the response range, the position of maximal occupancy, and sensitivity to MAFG-dependent competition. Because the intracellular concentrations and stoichiometric relationship of the available NRF2 and MAFG pools were not directly measured, these shifts describe relative behavior within the model and should not be interpreted as quantitative estimates of cellular concentration thresholds.

The model predicted that NRF1, NRF2, and NRF3 would show broadly similar MAFG-dependent occupancy profiles on the WT ARE (Fig. 6E), suggesting that partner-dependent complex formation may be shared across the NRF-sMAF family. We then organized the calculated WT NRF2-MAFG occupancy surface into four connected, model-defined concentration regimes (Fig. 6F). Within this calculated scheme, regime 1 represents a basal-like low-NRF2/low-MAFG condition. An acute increase in NRF2 in response to stress moves the system from regime 1 to regime 2, whereas subsequent MAFG induction during sustained stress moves it from regime 2 to regime 3. Following stress removal, rapid NRF2 turnover [49] with transiently persistent MAFG moves the system from regime 3 to regime 4, before MAFG returns toward the basal range. This concentration-regime map also defines an experimentally testable comparison across the MAFG dimension: at lower NRF2, a MAFG titration samples the axis connecting regimes 1 and 4, whereas at higher NRF2 it samples the axis connecting regimes 2 and 3. The model predicts that these two MAFG-dependent responses will differ. The arrows in Fig. 6F illustrate possible physiological trajectories across the predicted occupancy surface rather than reconstructed endogenous time courses or experimentally defined regime boundaries [13], [50].

We next tested whether the predicted biphasic dependence on MAFG could be detected in cells. HEK-293 cells were transiently transfected with a firefly luciferase reporter driven by the ARE sequence CCTGAGTCACG, together with a fixed amount of NRF2-K7, a degradation-resistant NRF2 mutant in which seven lysine residues are replaced with alanines, and increasing amounts of full-length MAFG (Fig. 6G). NRF2-K7 alone increased reporter activity relative to the empty-vector control, and addition of a low amount of MAFG further enhanced the response. Reporter activity exhibited a biphasic, dose-dependent response, reaching its maximum at 5 ng MAFG and then declining through 10–80 ng MAFG (ordered-dose quadratic contrast, P < 0.0001; Fig. 6G). These results demonstrate that MAFG exerts a biphasic effect on NRF2-dependent transcription.

Finally, we examined how derived apparent residence-time parameters shape occupancy within a biologically relevant finite-time observation window. In a simplified two-state sampling model, targets were assigned the same equilibrium occupancy but different residence half-lives (Fig. 6H). Over a 1-h observation window, shorter residence times produced repeated binding and dissociation events and therefore reduced variation in time-averaged occupancy. At longer residence times, fewer sampling events occurred within the same observation window, increasing occupancy variation over the finite observation period despite unchanged equilibrium occupancy. The derived apparent WT NRF2-MAFG-ARE residence half-life of approximately 16.2 s fell within the rapid-sampling range of this simplified calculation. Thus, residence time may influence not only DNA-bound complex stability but also how frequently ARE-bound complexes are resampled over biologically relevant time intervals.

## 3 Discussion

Together, these findings establish a hierarchical mechanism for NRF-sMAF-ARE complex formation. NRF-sMAF-ARE occupancy emerges from sequential control of dimer formation, DNA binding, and complex exchange rather than NRF abundance or ARE sequence alone. Dimer compatibility first restricts the complexes available for DNA binding, ARE sequence differentially stabilizes the alternative DNA-bound complexes, and exchange between pre-existing complexes allows conversion between DNA-bound complexes. This mechanism provides a biochemical explanation for how changes in NRF and sMAF abundance can be translated into distinct, sequence-dependent occupancy outcomes.

The first step in hierarchical complex formation is partner compatibility, which defines the dimer species available for ARE binding. Our NanoBRET matrix showed that NRF-sMAF heterodimers and sMAF-sMAF dimers define the dimer species available for ARE binding, whereas NRF-NRF interactions were not detected under the measured conditions. The absence of detectable NRF-NRF pairing, together with robust NRF-sMAF and sMAF-sMAF interactions, indicates that partner compatibility acts as an upstream specificity filter before DNA is encountered. NRF1 and NRF2 formed particularly strong heterodimers with MAFG and MAFK, while NRF3 occupied a weaker interaction tier, and sMAF homodimers were broadly detectable. NRF proteins therefore do not simply compete for AREs as independent DNA-binding factors. Their formation of DNA-bound complexes depends on the abundance and affinity of available sMAF partners. Differences among NRF1, NRF2, and NRF3 in their affinities for individual sMAFs may consequently influence which NRF family member gains access to AREs under different expression conditions.

ARE sequence is the second determinant in hierarchical complex formation and changes the relative occupancy of the detected dimer species. The same ARE substitutions produced different affinity effects depending on the dimer context. Thus, sequence sensitivity was a property of the dimer-DNA combination rather than either DNA sequence or dimer identity alone. Mutations within the core ARE region strongly disrupted NRF-MAFG recognition, while other positions produced more moderate but still measurable effects. These findings argue against treating AREs as a single interchangeable motif class. Instead, individual ARE sequences may be intrinsically biased toward different NRF-sMAF or sMAF-sMAF complexes, allowing sequence variation to influence not only overall binding strength but also the composition of the DNA-bound population.

DNA-bound complex stability is the third determinant in hierarchical complex formation. ARE binding site sequence affected not only apparent affinity, but also derived residence-time parameters, with sequence discrimination reflected strongly in dissociation-related parameters. This suggests that NRF-MAFG-ARE recognition is shaped by the stability of the DNA-bound complex after initial encounter, rather than only by differences in initial capture. The apparent association rate decreased by approximately 1.45- to 6.33-fold across single-base variants, while the largest apparent dissociation rate was approximately 14.7-fold above WT. Both processes therefore contribute to sequence sensitivity, while the larger changes in derived apparent k_off_ for several core substitutions support a prominent role for DNA-bound complex stability. Different AREs may therefore distinguish among complexes not only by permitting binding but also by determining how long the resulting DNA-bound complex persists under the measured conditions.

Reversible exchange between competing DNA-bound complexes is the fourth step in hierarchical complex formation. NRF2-MAFG-ARE complexes formed from pre-existing MAFG-MAFG-ARE complexes, indicating that a sMAF-occupied ARE need not represent a static repressive endpoint. Instead, it can serve as an intermediate from which an NRF-sMAF-ARE complex forms. The coupled dimerization and DNA-binding network may therefore permit exchange among competing DNA-bound complexes without requiring complete dissociation and reassembly exclusively from free proteins and DNA. The slower phase of NRF2-MAFG-ARE association may reflect such exchange coupled to complex formation, although the microscopic species responsible for this phase remain unresolved.

Recent biochemical work using preassembled NRF2-MAFG and BACH1-MAFG DNA-binding-domain heterodimers showed that NRF2-MAFG can replace a DNA-bound BACH1-MAFG complex at CsMBE sequences and that displacement is more efficient when the incoming factor is supplied as a preformed heterodimer rather than as an isolated bZIP domain [51]. These observations provide complementary support for a complex-exchange network in which a sMAF-containing DNA-bound complex is not necessarily a terminal complex. Within the hierarchical mechanism of complex formation, BACH1-MAFG represents an additional partner-defined, transcriptionally repressive DNA-bound complex, and changes in relative partner abundance, pre-formation of heterodimers, and redox-dependent DNA-binding competence may shift the relative occupancy of BACH1-MAFG and NRF2-MAFG complexes. Because the study used tethered DNA-binding-domain heterodimers in a reconstituted EMSA system, the molecular pathway and kinetics of this transition in cells remain to be established.

The occupancy model provides a quantitative biochemical explanation for the experimentally observed biphasic effect of MAFG in NRF2 signaling. At a fixed NRF2 input, increasing MAFG initially promotes formation of NRF2-MAFG heterodimers and supports ARE-dependent transcription, whereas further increases favor competing MAFG-containing complexes and constrain NRF-sMAF-ARE occupancy. The cellular reporter measurements provide functional evidence that this concentration-dependent complex balance can shape NRF2-dependent transcription. In the transient reporter, lower MAFG expression inputs supported the NRF2-dependent response, whereas higher inputs constrained it, reproducing the biphasic direction predicted by the occupancy calculation. These reporter conditions therefore test the response across graded MAFG expression inputs without assigning absolute intracellular protein concentrations or NRF2:MAFG ratios. The resulting biphasic response provides cellular functional support for the complex-balance mechanism defined biochemically and predicted computationally, although the reporter readout does not itself distinguish the individual DNA-bound complexes contributing to the response.

The derived apparent residence times also suggest that ARE-bound complexes may differ in how frequently they are resampled over finite biological intervals. In the simplified sampling analysis, shorter-lived modeled DNA-bound complexes underwent more binding and dissociation cycles and showed less variation in time-averaged occupancy than longer-lived modeled DNA-bound complexes at the same equilibrium occupancy. The derived apparent wild-type NRF2-MAFG-ARE residence half-life of approximately 16.2 s fell within this rapid-sampling range. Although this calculation does not model transcription directly, it illustrates why sites with similar equilibrium occupancy could nevertheless differ in temporal stability and responsiveness.

More broadly, the shared MAFG-dependent response predicted across NRF1, NRF2, and NRF3 suggests that hierarchical control of complex formation may apply broadly across heterodimeric bZIP transcription-factor networks. In this mechanism, partner compatibility defines the dimer species available for ARE binding, DNA sequence changes the relative occupancy of DNA-bound NRF-sMAF and sMAF-sMAF complexes, DNA-bound complex stability determines persistence, and reversible exchange connects competing DNA-bound complexes. Therefore, the NRF-sMAF-ARE system provides an experimentally resolved example of this combination of dimer formation, sequence-dependent DNA binding, complex stability, and exchange, but whether related bZIP networks use the same sequence of molecular constraints remains to be determined.

Most mechanistic experiments were performed using a purified system and therefore do not incorporate chromatin context, cofactors, post-translational regulation, NRF turnover, or dynamic cellular expression. The cellular reporter assays extend the biochemical and computational mechanism to a functional readout, although they do not directly resolve the individual NRF2-MAFG and MAFG-MAFG complexes or reconstruct endogenous concentration boundaries. Their responses to graded MAFG expression inputs are nevertheless consistent with the abundance-dependent balance among MAFG-containing complexes predicted by the experimentally parameterized model, although the experiments do not assign this response to absolute intracellular protein concentrations or stoichiometric boundaries. In addition, each NanoBRET configuration reports a predefined donor-acceptor proximity signal within a coupled dimerization and DNA-binding network. The apparent dissociation constants and kinetic parameters should therefore be interpreted as condition-dependent descriptors of measured NanoBRET readouts rather than elementary microscopic constants. The biphasic association kinetics indicate an additional transition during complex formation, but the molecular identity of the slower phase remains unresolved and will require simultaneous monitoring of multiple molecular interactions. Finally, whether the broader principle of hierarchical complex formation applies outside the NRF-sMAF system remains to be tested.

Together, these findings establish a hierarchical mechanism of complex formation in which partner compatibility, dimer-dependent DNA recognition, DNA-bound complex stability, reversible exchange between DNA-bound complexes, and relative NRF-sMAF abundance collectively determine NRF-sMAF-ARE occupancy. This mechanism extends the biochemical interpretation of NRF signaling beyond NRF activation and ARE availability by showing how relative NRF and sMAF abundance, dimer identity, and ARE sequence jointly determine NRF-sMAF-ARE occupancy. The biphasic MAFG dose response in the transient reporter directly demonstrates, in that cellular context, that lower and higher MAFG expression inputs can respectively support and constrain NRF2-dependent transcription. More broadly, these findings suggest that transcription-factor occupancy by activating complexes may emerge from competition among molecular complexes rather than from protein abundance or DNA affinity alone. Whether similar principles of hierarchical complex formation govern other heterodimeric transcription-factor systems remains an important question for future investigation.

## 4 Materials and methods

Detailed methods are provided in the Supplemental Information. Recombinant NRF and sMAF constructs were expressed and purified as N-terminal NanoLuc donor fusions or NbALFA fusion proteins for acceptor labeling. In the acceptor configuration, protein-fused NbALFA bound a fluorophore-labeled ALFA peptide, thereby introducing the acceptor dye without direct chemical labeling of the target protein. Protein-protein NanoBRET assays used ALFA-ATTO490LS to test NRF-NRF, NRF-sMAF, and sMAF-sMAF interactions in reciprocal donor-acceptor configurations, while protein-DNA NanoBRET assays used ATTO590-labeled ARE to quantify protein-DNA association in the corresponding NRF-MAFG and MAFG-MAFG dimer contexts with unlabeled MAFG present. Real-time NanoBRET injection assays measured association kinetics and order-of-addition effects. Apparent equilibrium parameters were used to parameterize the simplified occupancy model, whereas derived residence-time effects were evaluated using a simplified kinetic sampling model.

## CRediT authorship contribution statement

**Yongyi Wei:** Writing – review & editing, Writing – original draft, Methodology, Investigation, Formal analysis, Conceptualization. **Lewis Alexander:** Writing – review & editing, Investigation. **Haofeng Ye:** Writing – review & editing, Methodology, Investigation. **Xiaoyi Zhu:** Methodology, Investigation. **Qiu Li:** Writing – review & editing, Methodology, Formal analysis. **Ke Wang:** Writing – review & editing, Methodology. **Zhaoyuan Sun:** Writing – review & editing, Methodology. **Dichun Huang:** Methodology, Validation. **Hendrik Luesch:** Writing – review & editing, Methodology. **Aikseng Ooi:** Writing – review & editing, Writing – original draft, Methodology, Investigation, Formal analysis. **Eli Chapman:** Writing – review & editing, Writing – original draft, Funding acquisition, Formal analysis. **Donna Zhang:** Writing – review & editing, Writing – original draft, Funding acquisition, Formal analysis, Conceptualization.

## Declaration of competing interest

The authors declare that they have no known competing financial interests or personal relationships that could have appeared to influence the work reported in this paper.

## Data Availability

Data will be made available on request.
