# Supplementary material for: Quantitative mapping of NRF-sMAF-ARE interactions reveals biphasic regulation of NRF2-dependent transcription by MAFG
Source: Redox Biol. 2026 Nov 5;98:104474. doi: 10.1016/j.redox.2026.104474 (PMC13763455; doi:10.1016/j.redox.2026.104474)
Supplement: Supplementary file 1 [file mmc2.pdf]

Quantitative mapping of NRF-sMAF-ARE interactions reveals biphasic regulation of NRF2-dependent transcription by MAFG

Yongyi Wei[1,2], Lewis Alexander[2,3], Haofeng Ye[1,2], Xiaoyi Zhu[1,2,4], Qiu Li[1,2], Ke Wang[1,2], Zhaoyuan Sun[5], Dichun Huang[1,2,4], Hendrik Luesch[5,6,7], Aikseng Ooi[2,6,8,*], Eli Chapman[2,3,6*], Donna Zhang[1,2,6,*]

[1]Department of Molecular Medicine, The Herbert Wertheim UF Scripps Institute for Biomedical Innovation and Technology, Jupiter, Florida, USA

[2]Center for Inflammation Science and Systems Medicine, The Herbert Wertheim UF Scripps Institute for Biomedical Innovation and Technology, Jupiter, Florida, USA

[3]Department of Pharmacology and Therapeutics, University of Florida, Florida, USA

[4]Department of Pharmacology and Toxicology, College of Pharmacy, University of Arizona, Tucson, Arizona, USA

[5]Department of Medicinal Chemistry and Center for Natural Products, Drug Discovery and Development (CNPD3), College of Pharmacy, University of Florida, Gainesville, Florida, USA.

[6]University of Florida Health Cancer Center, University of Florida, Gainesville, Florida, USA

[7]Program in Cancer and Stem Cell Biology, Duke-NUS Medical School, Singapore, Singapore.

[8]Department of Medicine, Division of Hematology Oncology, College of Medicine, University of Florida, Florida, USA

*To whom correspondence should be addressed. Email: zhangd1@ufl.edu; Email: chapmaneli@ufl.edu; Email: aikseng.ooi@medicine.ufl.edu

Table of Contents

Materials and Methods

Protein expression and purification 1
ALFA peptide labeling and spectral characterization 1
Labeled ARE preparation 2
NanoBRET assay development and validation 2
NanoBRET leucine-zipper dimerization assay 3
NanoBRET ARE-binding assay 4
Kinetic NanoBRET assay for ARE association 4
Data processing and curve fitting 7
Calculation of apparent kinetic parameters 8
Coupled-equilibrium modeling of ARE occupancy 10
Calculation of finite-time occupancy variability 12
Cellular ARE-reporter assays 12

Supporting Figures

Figure S1. Supporting characterization of the NanoBRET measurement framework 14
Figure S2. Analytical robustness of the NanoBRET readout across defined and equilibrium-dependent engagement readouts 15
Figure S3. Complete NRF–NRF interaction matrix shows no detectable homo- or heterotypic NRF dimerization under the measured conditions 16
Figure S4. Extended ARE sequence-dependent engagement profiles and competition validation of protein–DNA NanoBRET 17
Figure S5. Biphasic association kinetics provide evidence for coupling within NRF2–MAFG–ARE assembly 18
Figure S6. Extended association kinetics of WT and mutant ARE sequences 19
Figure S7. Raw NanoBRET reference signals for alternative NRF2–MAFG–ARE assembly-order measurements 20
Figure S8. Model-predicted ARE sequence-dependent sensitivity to MAFG-mediated constraint of NRF–MAFG–ARE occupancy 21

Supporting Tables

Table S1. Spectral parameters of donor–acceptor pairs used for NanoBRET evaluation 23
Table S2. Comprehensive quantification of NRF–MAF interaction affinities by NanoBRET 24
Table S3. Thermodynamic parameters of NRF–MAFG–ARE and MAFG–MAFG–ARE interactions measured by NanoBRET 25

Table S4. Comparison of kinetic fitting approaches for NRF–sMAF–ARE association traces .26
Table S5. Apparent kinetic parameters for ARE variants in NRF2–MAFG–ARE binding 26
Table S6. Key Resources Table 28
Table S7. Sequences and naming conventions of all ARE variants 29

Additional Supporting Data

Supporting Information Excel file: ARE-binding K_d fitting; leucine-zipper K_d fitting; ARE kinetic fitting; leucine-zipper kinetic fitting; and plasmid information 29

Materials and Methods

Key reagents, biological resources, oligonucleotides, software, and associated identifiers used in this study are summarized in Table S6.

Protein expression

Expression constructs were generated using the CIDAR Modular Cloning (MoClo) system. Coding sequences were codon-optimized for *E. coli,* synthesized as double-stranded gene fragments lacking internal Type IIS restriction sites (IDT), and assembled into a T7-driven MoClo expression backbone by Golden Gate assembly using BsaI and T4 DNA ligase. Assembly products were transformed into NEB Stable cells, sequence-verified by Sanger sequencing, and then transformed into SHuffle® T7 Express lysY *E. coli* for protein production. Detailed construct information, insert sequences, and oligonucleotide sequences are provided in Table S7 and Supporting Information Excel Sheet 5. A single colony was inoculated into 20 mL of 2xYT medium containing the appropriate antibiotic and grown overnight at 37°C with shaking. The culture was then transferred into 500 mL of fresh 2xYT medium with antibiotic and incubated with shaking until reaching mid-log phase. Protein expression was induced with 1 mM IPTG and allowed to proceed for 2 h at 37°C under aerobic shaking conditions. Cells were harvested by centrifugation and stored at −20°C until purification.

Protein purification

Cell pellets were resuspended in CB (20 mM Tris-HCl, pH 7.5–8.0, 300 mM NaCl, and 20 mM imidazole) supplemented with protease inhibitors. Lysates were prepared by sonication on ice and clarified by centrifugation. The supernatant was applied to Ni-NTA resin that had been equilibrated in the same buffer. Bound proteins were washed with 20-50 mM imidazole and eluted with 250 mM imidazole in CB buffer. Eluted fractions were analyzed by SDS–PAGE, pooled based on purity, and desalted into storage buffer. The storage buffer consisted of CB buffer supplemented with 0.1 mM DTT. Glycerol was added to a final concentration of 40% (v/v), and aliquots were stored at -20°C. SDS–PAGE analysis of purified proteins is shown in Figure S1. For the tag-cleavage control experiments, the N-terminal His tag was removed from His-3C-Nluc-MAFG using HRV 3C protease to generate Nluc-MAFG, whereas the N-terminal MBP tag was removed from MBP-NRF2 using Factor Xa to generate untagged full-length NRF2. The resulting proteins were used in the subsequent ARE-binding measurements.

Peptide labeling

ALFA peptides containing an N- or C-terminal lysine residue were synthesized by GenScript (Piscataway, NJ, USA) at >98% purity. Peptides were supplied in lyophilized form and dissolved in PBS to the desired stock concentration immediately before use. NHS-ester dyes were obtained from Lumiprobe (Cy3, Cy5, Cy5.5, AF488, AF568), Sigma-Aldrich (ATTO590), and ATTO-TEC (ATTO430, ATTO490LS). All dyes were freshly prepared in anhydrous DMF at 10 mM. For peptide-dye conjugation, 50 µL of ALFA peptide solution was mixed with 5.5 µL of each NHS-ester dye, corresponding to a 1:1.1 peptide:dye molar ratio in PBS. Reactions were incubated for 2 h at room temperature with gentle agitation. Excess NHS-ester dye was quenched by the addition of 44.5 µL of 10 mM Tris buffer (pH 8.0) to yield a final dye concentration of 0.5 mM, followed by

incubation for 2 hours room temperature. Because the NanoBRET assay is tolerant to low levels of free dye under the assay conditions used here, the reaction mixtures were used directly as labeled-peptide stocks without further purification. Quenched-dye controls were included to monitor any non-specific contribution from free dye. Conjugates were aliquoted and stored at -20°C protected from light. For negative control dyes, 5.5 µL of the 10 mM NHS-ester dye was mixed with 94.5 µL of 10 mM Tris buffer (pH 8.0) to generate a quenched-dye stock at 0.55 mM. This slightly higher concentration was chosen to match the total dye content present in the labeled samples, because peptide labeling was performed at a 1:1.1 peptide:dye molar ratio. Consequently, the resulting labeled-peptide stock contains a total dye concentration corresponding to this 10% excess. The solution was incubated for 2 h at room temperature and used without further purification. Aliquots were stored at -20°C in the dark.

Spectral characterization

To assess the spectral compatibility of Nluc with each dye, absorbance spectra of donor proteins and dye-peptide conjugates were recorded on a CLARIOstar Plus plate reader in transparent 96-well plates at room temperature. Emission spectra of donor luminescence and acceptor fluorescence were then acquired on the same plate reader using white 96-well plates, the Nluc substrate, and appropriate emission bandwidth settings. Spectral overlap integrals J(λ), predicted Förster radii, and effective Stokes shifts were calculated by numerical integration using custom R routines with a wavelength step size of 1 nm.

Labeled ARE preparation (ATTO590-ARE duplex)

ATTO590-labeled ARE probes were assembled from three oligonucleotides: a 40-bp ARE-containing forward strand, a fixed 20-bp 5′-ATTO590-labeled sensor strand, and a 60-bp reverse strand complementary to both forward components. The 60-bp reverse strand contained a 40-bp region complementary to the ARE-containing strand and a 20-bp region complementary to the fixed labeled sensor strand. Oligonucleotides were obtained from IDT at 100 µM in Tris buffer. For each preparation, 10 µL of the 40-bp ARE forward strand, 10 µL of the 20-bp 5′-ATTO590-labeled sensor strand, 10 µL of the 60-bp reverse strand, and 10 µL of 10 mM Tris were combined. The mixture was heated to 75°C for 5 min and cooled to 4°C at 0.1°C/s to generate the assembled duplex. The resulting ATTO590-ARE stock had a final concentration of 25 µM. For each ARE mutant, the 40-bp ARE forward strand and the corresponding 40-bp complementary region of the 60-bp reverse strand were changed to introduce the desired sequence variation. The 20-bp ATTO590-labeled sensor strand and its corresponding complementary region remained unchanged across all ARE variants, maintaining a constant fluorophore position and sensor sequence while varying only the 40-bp ARE region (Table S7).

NanoBRET assays

All NanoBRET assays were conducted in CBT buffer supplemented with 0.5 mM TCEP at room temperature unless otherwise noted. Furimazine was supplied as a 1 mM stock and diluted directly into assay wells to the specified final concentration. Luminescence was recorded using a 420 nm donor channel (20 nm bandwidth), a 620 nm acceptor channel for ATTO490LS (100 nm bandwidth), and a 660 nm acceptor channel for ATTO590 (100 nm bandwidth). BRET signals were calculated as the acceptor-to-donor emission ratio (mBRET).

Dye-selection NanoBRET screen (NbALFA-ALFA panel)

For initial dye evaluation, NanoBRET reactions contained NbALFA-Nluc at 10 pM and ALFA peptide labeled with individual dyes (Cy3, Cy5, Cy5.5, AF488, AF568, ATTO430, ATTO490LS, ATTO590) at 10 nM in CBT buffer. Reactions were assembled in white 96-well plates at a final volume of 100 µL per well and incubated for 30 min at room temperature. Furimazine was then added at 1 µL per well to reach a final concentration of 10 µM, and NanoBRET was read within 2 min. Donor quenching, acceptor enhancement, and overall mBRET window were compared across dyes to select ATTO490LS and ATTO590 as the optimal acceptors for subsequent protein-protein interaction (PPI) and stability assays.

NanoBRET PPI and assay-stability format (NbALFA-Nluc/ALFA-ATTO490LS)

For assay-robustness experiments, NanoBRET reactions were assembled using NbALFA-Nluc (10 pM) and ALFA-ATTO490LS peptide (10 nM) in CBT buffer with 0.5 mM TCEP. Unless otherwise specified, reactions were dispensed into white 96-well plates at 100 µL per well, incubated for 30 min, and then supplemented with furimazine (1 µL per well, final 10 µM). NanoBRET was recorded at defined time points to monitor signal stability and dynamic range.

Competition and engagement controls

To confirm that the NanoBRET signal reflected specific NbALFA-ALFA engagement, labeled and unlabeled ALFA peptides were premixed at defined labeled fractions (0%, 50%, 100%) at a fixed total peptide concentration of 10 nM and incubated with NbALFA-Nluc (10 pM) for 60 min. Donor and acceptor signals were measured as above, and mBRET was analyzed as a function of labeled fraction. For the defined engagement references, the labeled fraction was varied from 0% to 100% while the total ALFA peptide concentration was held at 10 nM.

Robustness tests

To assess volume and plate-format robustness, reactions were run in 96-well plates at volumes ranging from 10 to 200 µL and in 384-well plates at volumes ranging from 10 to 50 µL, using identical donor, acceptor, and substrate conditions, and NanoBRET was quantified as the corrected 620/420 emission ratio. Donor-concentration robustness was evaluated by titrating NbALFA-Nluc from 1 pM to 3 nM at fixed ALFA-ATTO490LS (10 nM) and quantifying effects on mBRET, standard deviation, and %CV. Acceptor-concentration robustness was assessed by titrating ALFA-ATTO490LS from 0.001 nM to 300 nM at fixed donor concentration (10 pM).

NanoBRET leucine-zipper dimerization assay

NanoBRET measurements for NRF-sMAF leucine-zipper dimerization were carried out in CBT buffer supplemented with 0.5 mM TCEP at room temperature. Donor constructs consisted of N-terminal Nluc fusions of NRF1, NRF2, NRF3, MAFG, MAFF, or MAFK. Acceptor partners were introduced through NbALFA bound to ALFA-ATTO490LS. Luminescence was collected using a 420 nm donor channel and a 620 nm acceptor channel, and BRET was expressed as the corrected 620/420 nm emission ratio. For each donor-acceptor pair, Nluc-tagged donor was used at 10 pM, and 350 nM ALFA-ATTO490LS was titrated with NbALFA-protein over a range of 0-316 nM in CBT buffer. Reactions were assembled in white 96-well plates at a final volume of 100 µL

per well and incubated for 60 min prior to substrate addition. Furimazine (1 μL of a 1 mM stock; final 10 μM) was added immediately before reading, and mBRET values were collected within 2 min. This assay format was applied across the full NRF/sMAF panel to generate a complete interaction matrix. mBRET versus acceptor concentration curves were fit to a one-site binding isotherm to obtain apparent K_d values for each dimer combination. These values are reported as apparent because they reflect effective binding under the defined assay conditions rather than isolated microscopic two-component interactions.

NanoBRET ARE-binding assay

NanoBRET measurements for ARE binding were carried out in CBT buffer supplemented with 0.5 mM TCEP at room temperature. Donor constructs consisted of N-terminal Nluc fusions of NRF1, NRF2, NRF3, or full-length MAFG. Unlabeled MAFG (3 nM) was included in all reactions to favor formation of the relevant leucine-zipper dimer before DNA engagement. The acceptor consisted of an ATTO590-labeled composite duplex containing a 40-bp NQO1 ARE region and a fixed 20-bp sensor region. Luminescence was collected using a 420 nm donor channel and a 660 nm acceptor channel, and NanoBRET was expressed as the 660/420 nm emission ratio. For each donor-ARE condition (NRF1/2/3 or MAFG), Nluc-tagged donor was used at 10 pM, unlabeled MAFG at 3 nM, and ATTO590-ARE was titrated over the desired concentration range (typically 0–316 nM) in CBT buffer. Reactions were assembled in white 96-well plates at a final volume of 100 μL per well and incubated for 60 min prior to substrate addition. Furimazine (1 μL of a 1 mM stock; final 10 μM) was added immediately before reading, and 660/420 nm emission ratio values were collected within 2 min. This assay format was applied across NRF1, NRF2, NRF3, and MAFG for WT and mutant ARE sequences. Binding was measured in the presence of 3 nM unlabeled MAFG to favor formation of the relevant dimeric species before DNA engagement. Because competing equilibria remain possible under these conditions, the resulting values are reported as apparent K_d values. Binding curves were normalized to WT NQO1 ARE, and mBRET versus ATTO590-ARE concentration was fit to a one-site binding isotherm.

Kinetic NanoBRET assay for ARE association

The kinetic NanoBRET assay was divided into two acquisition conditions based on temporal sampling and signal-intensity requirements rather than on different binding mechanisms. The rapid-acquisition condition was designed to provide sufficient signal intensity for high-frequency scanning, whereas the extended-acquisition condition was designed to support longer-duration monitoring while limiting substrate consumption. Because the two conditions used different donor concentrations and acquisition settings, kinetic traces were normalized to matched negative and pre-equilibrated reference conditions measured under the corresponding assay settings. Thus, the distinction between the two acquisition conditions was technical rather than mechanistic.

<u>Condition 1: Rapid-acquisition condition</u>

Association kinetics were measured in a real-time NanoBRET format under conditions optimized to capture rapid association behavior within a 0-100 s acquisition window at 0.1 s per scan. Reactions were assembled in CBT buffer supplemented with 0.5 mM TCEP. Unless otherwise specified, Nluc-ΔNRF2 was used as the donor construct at a final concentration of 300 pM, unlabeled MAFG at 3 nM, and ATTO590-ARE as the acceptor at 1 nM. For each measurement,

50 µL of a 2× protein mixture was dispensed into each well of a white 96-well plate and equilibrated for 1 h before kinetic testing. At the start of the measurement, 50 µL of a 2× mixture containing ATTO590-ARE and substrate was injected using the plate reader injector to initiate the reaction. Signals were collected every 0.1 s for 100 s. To minimize acquisition time and avoid filter switching during rapid kinetic measurements, only the acceptor emission channel at 660 nm with 100 nm bandwidth was monitored. Signals were normalized using two stable reference conditions measured under the same NanoBRET settings. In the negative condition, wells contained Nluc-ΔNRF2 and unlabeled MAFG without ATTO590-ARE, and substrate was injected at the start of acquisition. In the balanced condition, wells contained Nluc-ΔNRF2, unlabeled MAFG, and ATTO590-ARE pre-equilibrated for 1 h before measurement, and substrate alone was injected at the start of acquisition. For the pre-occupied-ARE exchange experiment (Fig. 5D), an NRF2/MAFG mixture and a MAFG/ARE mixture were preincubated separately before kinetic acquisition. A 50 µL 2× mixture containing Nluc-ΔNRF2 and unlabeled MAFG and a separate 50 µL 2× mixture containing ATTO590-labeled ARE and unlabeled MAFG were each incubated for 1 h to approach the equilibrium distribution generated by their components. Furimazine was added to the MAFG/ARE mixture immediately before it was injected into the Nluc-ΔNRF2/MAFG mixture. The design changes whether ARE had equilibrated with MAFG before the full composition was established; it does not assume a single molecular species in either mixture, and protein-partner reorganization and protein–DNA engagement remain coupled after mixing. The final reaction contained 300 pM Nluc-ΔNRF2, 3 nM total unlabeled MAFG, and 1 nM ATTO590-ARE. Acceptor emission was recorded every 0.1 s for 100 s and normalized to the matched reference conditions. Development of NRF2–ARE proximity reported NRF2-MAFG-ARE formation from an ARE population previously equilibrated with MAFG.

<u>Condition 2: Extended-acquisition condition</u>

Association kinetics were measured in a real-time NanoBRET format under conditions optimized to monitor extended association behavior within a 0-1000 s acquisition window at 1 s per scan. Reactions were assembled in CBT buffer supplemented with 0.5 mM TCEP. Unless otherwise specified, Nluc-ΔNRF2 was used as the donor construct at a final concentration of 10 pM, unlabeled MAFG at 3 nM, and ATTO590-ARE as the acceptor at 1 nM. To prevent initiation of the luminescence reaction before kinetic acquisition, assay components were separated prior to injection so that substrate was added only at the start of measurement. For the alternative-order experiment (Fig. 5A), final NRF2, MAFG, and ARE concentrations and the NRF2–ARE readout were held constant while the preincubated mixture and injected component differed. (1) NRF2-addition route: MAFG and ATTO590-ARE were preincubated for 1 h before Nluc-ΔNRF2 and substrate were injected. (2) MAFG-addition route: Nluc-ΔNRF2 and ATTO590-ARE were preincubated for 1 h before unlabeled MAFG and substrate were injected. Each mixture was allowed to approach the equilibrium distribution generated by its components without assuming a single molecular species. The route labels identify the injected component, not an exclusive reaction; protein–protein and protein–DNA processes could both contribute after mixing. (3) Balanced reference: Nluc-ΔNRF2, MAFG, and ATTO590-ARE were preincubated for 1 h before substrate alone was injected. (4) Negative reference: Nluc-ΔNRF2 and MAFG were preincubated for 1 h before substrate was injected. Reference settings otherwise matched Condition 1 except that the donor concentration was 10 pM.

Data processing

All reported binding constants represent apparent values derived under defined experimental conditions in the presence of coupled equilibria. Raw plate-reader files were imported into Excel. Background subtraction was performed using wells containing buffer plus substrate only. For each dye, the signal was normalized to the Nluc-only control or to a designated standard well. Variability was reported as mean ± SD across three independent experiments.

Linearity and Z′ calculation

To quantify linearity between binding fraction and mBRET:

- Labeled/unlabeled mixtures covering 0–100% labeled fractions were prepared.
- For each fraction, 8 replicate wells were measured.

Linear regression was performed (mBRET vs % labeled), and Z' factors were computed using:

$$Z' = 1 - \frac{3(\sigma_p + \sigma_n)}{|\mu_p - \mu_n|}$$

where μ_p and μ_n are the means, and σ_p and σ_n are the standard deviations of the positive (e.g., 100% labeled) and negative (e.g., 0% labeled) control populations, respectively. Z' values were determined separately for distinguishing 0% controls and specific target binding levels.

Structural rationale for assay design

Sequence alignment and domain annotation

Protein sequences for NRF1, NRF2, NRF3, MAFG, MAFF, and MAFK were retrieved from UniProt (accession IDs Q16656, Q16236, Q9Y4A8, O15525, Q9ULX9, and O60675, respectively). Multiple sequence alignments were generated using Clustal Omega with default parameters (1). Heptad repeat positions (a-g), basic DNA-binding regions, and Neh1/Neh3 domains were annotated manually based on conserved sequence motifs and published domain boundaries. Structural predictions were obtained by sequence alignment and by using AlphaFold 3 through the AlphaFold Server interface (2).

mBRET calculation and curve fitting

For each well, raw luminescence was recorded in both donor and acceptor channels, and buffer-only wells were used to subtract instrument background from each channel. Because the acceptor channel has a wider bandwidth than the donor channel, control wells containing Nluc together with quenched free dye (no acceptor-labeled binding partner) were included in every plate to define the baseline donor bleed-through into the acceptor channel. The initial corrected acceptor/donor ratio was computed as:

$$\frac{I_{acceptor}}{I_{donor}}\text{-}R_{baseline},$$

where Iacceptor and Idonor are background-subtracted intensities, and Rbaseline is the mean acceptor/donor ratio obtained from control wells. To express this signal in standardized milliBRET units, mBRET values were calculated as:

$$\text{mBRET}=1000\times\left(\frac{I_{acceptor}}{I_{donor}}\text{-}R_{baseline}\right).$$

Binding curves (mBRET vs. acceptor concentration) were fit to a single-site binding isotherm:

$$B=\frac{B_{max}[A]}{K_d+[A]},$$

using nonlinear regression in R (nls). For each sample, best-fit Bmax and K_d values, their 95% profile-likelihood confidence limits, and R^2 values were obtained from independent fits and exported for downstream analysis.

Kinetic data normalization

Raw kinetic traces were exported to Prism for analysis. For each binding curve, luminescence signals were normalized using the negative and balanced reference conditions according to:

$$S(t)=\frac{I(t)-I_{neg}(t)}{I_{bal}(t)-I_{neg}(t)}\times 100\%$$

where S(t) is the normalized NanoBRET signal (%) at time t, I(t) is the measured luminescence intensity, I_{neg} is the intensity of the negative control (no acceptor), and I_{bal} is the intensity of the balanced (equilibrium) condition.

Kinetic curve fitting

<u>DNA binding kinetics</u>

Normalized association curves were fitted using single-exponential and bi-exponential association equations.

<u>Single-exponential association</u>

$$S(t)=S_0+\Delta S\left(1-e^{-k_{obs}t}\right)$$

where S(t) is the normalized signal at time t, S_0 is the post-injection baseline, ΔS is the binding-dependent amplitude, and k_{obs} is the apparent association rate constant at the given concentration of labeled ARE.

Bi-exponential association

$$S(t) = S_0 + \Delta S_1\left(1-e^{-k_1 t}\right) + \Delta S_2\left(1-e^{-k_2 t}\right)$$

where S_0 is the baseline signal, ΔS_1 and ΔS_2 are the amplitudes of the fast and slow kinetic phases, and k_1 and k_2 are the corresponding observed rate constants.

Leucine zipper exchange kinetics

The leucine-zipper exchange kinetic curves were adequately described by a single-exponential association equation; therefore, bi-exponential fitting was not pursued for this condition.

Calculation of apparent kinetic parameters

Association kinetics were analyzed using $k_{obs,fast}$, the observed rate constant of the rapid kinetic phase obtained from exponential fitting of NanoBRET time courses. For operational comparison of this rapid association-related component, $k_{obs,fast}$ was analyzed using the pseudo-first-order relationship:

$$k_{obs} = k_{on}[ARE] + k_{off}$$

where $[ARE]$ is the final concentration of labeled ARE in the kinetic assay. Because $k_{obs,fast}$ was extracted from pair-resolved NanoBRET traces within a coupled assembly system, the resulting kinetic parameters are interpreted as apparent effective parameters rather than elementary microscopic rate constants.

Method 1: Estimation of apparent k_{on} from concentration dependence

Values of $k_{obs,fast}$ were determined across a series of ARE concentrations and plotted as a function of $[ARE]$. Linear regression was performed according to:

$$k_{obs,fast} = k_{on}[ARE] + k_{off}$$

The slope of the regression was used to estimate the apparent association rate constant k_{on}, whereas the y-intercept represents the apparent contribution of k_{off}. The highest ARE concentration was excluded from the regression because the rapid association kinetics approached the temporal resolution limit of the assay, resulting in underestimation of $k_{obs,fast}$.

Method 2: Calculation of k_{on} from $k_{obs,fast}$ and K_d

For datasets in which a complete concentration-dependent kinetic series was not available, including ARE mutant measurements, apparent k_{on} was calculated by combining $k_{obs,fast}$ measured

at a fixed ARE concentration with the independently determined apparent equilibrium dissociation constant K_d:

$$k_{on} = \frac{k_{obs,fast}}{[ARE] + K_d}$$

This relationship follows from the combination of:

$$K_d = \frac{k_{off}}{k_{on}} \text{ and } k_{obs,fast} = k_{on}[ARE] + k_{off}$$

All concentrations correspond to the final concentrations present in the kinetic assay.

Treatment of multiphasic kinetics

The bi-exponential behavior indicated that a simple one-step model did not describe the complete NRF2-MAFG-ARE reaction. Fitting separated fast and slow components mathematically, not physical species or microscopic reactions. Based on its ARE-concentration dependence, the fast component ($k_{obs,fast}$) was interpreted as being dominated by DNA engagement and was used to compare apparent association parameters. The slower component was analyzed as a candidate protein-partner reorganization or exchange process and was not used to derive apparent k_{on}. These assignments are operational rather than exclusive.

For measurements initiated by MAFG or NRF2 addition, the concentration term was defined using the relevant binary NRF2–MAFG interaction. Because MAFG was present at 3 nM and donor-labeled NRF2 at 10 pM, the NanoBRET signal primarily reports the donor-labeled NRF2 population. Apparent k_{on} for the MAFG-dependent calculation was estimated using the equation below. This operational protein-partner assembly/exchange scale does not imply exclusively protein–protein kinetics.

$$k_{on} = \frac{k_{obs}}{[MAFG] + K_{d,binary}}$$

where [MAFG] is the final MAFG concentration in the assay and $K_{d,binary}$ is the apparent dissociation constant measured for the binary NRF2–MAFG interaction.

Calculation of the apparent dissociation rate constant k_{off}

For mutant comparisons and other datasets analyzed using Method 2, apparent k_{on} and k_{off} were not independently fitted but were derived from $k_{obs,fast}$ and the independently measured apparent K_d at a fixed ARE concentration according to:

$$k_{on} = \frac{k_{obs,fast}}{C + K_d}, k_{off} = \frac{k_{obs,fast}K_d}{C + K_d}.$$

where C is the final ARE concentration in the kinetic assay. For derived parameters, uncertainty was estimated by first-order error propagation from the fitting uncertainties in $k_{obs,fast}$ and K_d, and is reported as an approximate 95% limit.

Parameters from MAFG- or NRF2-initiated measurements were calculated using the protein–protein equilibrium and are reported on an operational protein-partner assembly/exchange scale. Parameters calculated from ARE concentration and the independently measured protein–DNA dissociation constant are reported on an operational ARE-engagement/remodeling scale. Both measurements arise from the same coupled protein–protein/protein–DNA network, and neither isolates a single microscopic reaction. The two ranges are therefore not estimates of the same elementary rate constant. Their marked difference in magnitude is consistent with the different equilibrium constraints and calculation definitions rather than a unit-conversion or numerical-scaling difference.

As an independent approach, k_{off} can also be estimated from the pseudo-first-order relationship:

$$k_{obs,fast} = k_{on}[\mathrm{ARE}] + k_{off}.$$

Fitting $k_{obs,fast}$ as a function of $[\mathrm{ARE}]$ yields k_{off} as the y-intercept. Although both approaches produced k_{off} estimates within the same order of magnitude, the intercept-based method was highly sensitive to low-concentration data points, which had large standard errors and resulted in unstable k_{off} estimates. Therefore, intercept-derived values were not used in the final analysis and are presented only for methodological comparison.

For comparative analysis, parameters were transformed into their logarithmic forms (pK_d, $-pk_{on}$, and pk_{off}) prior to computing differences relative to WT human NQO1 ARE. Because higher corresponds to faster association—opposite the direction of pK_d and pk_{off}—association-rate terms were reported as $-pk_{on}$ to maintain consistent interpretation across parameters. Differences relative to WT were expressed as ΔpK_d, $\Delta\text{-}pk_{on}$, and Δpk_{off}, representing log-scale shifts in affinity, association, and dissociation, respectively.

$$pk_{on} = -\log_{10}(k_{on})$$

$$pk_{off} = -\log_{10}(k_{off})$$

Coupled-equilibrium modeling of ARE occupancy

We used a coupled-equilibrium model to calculate ARE occupancy from the experimentally determined apparent dissociation constants. The model included NRF–sMAF heterodimerization and sMAF–sMAF homodimerization, followed by binding of either dimer to ARE. The apparent protein–DNA K_d values were used as effective occupancy-weighting parameters for the measured dimer-associated ARE-bound complexes and were not interpreted as isolated microscopic dimer–DNA equilibrium constants. Accordingly, the calculated occupancy represents an empirically parameterized occupancy framework rather than an exact microscopic reconstruction of the full equilibrium network.

At equilibrium, NRF–sMAF and sMAF–sMAF dimer concentrations were defined by:

$$K_{d,\mathrm{NRF-sMAF}} = \frac{[\mathrm{NRF}][\mathrm{sMAF}]}{[\mathrm{NRF-sMAF}]}$$
$$K_{d,\mathrm{sMAF-sMA}} = \frac{[\mathrm{sMAF}]^2}{[\mathrm{sMAF-sMAF}]}$$

subject to the mass-balance relationships:

$$[\mathrm{NRF}]_\mathrm{T} = [\mathrm{NRF}] + [\mathrm{NRF-sMAF}]$$
$$[\mathrm{sMAF}]_\mathrm{T} = [\mathrm{sMAF}] + [\mathrm{NRF-sMAF}] + 2[\mathrm{sMAF-sMAF}]$$

The coupled equations were solved for the non-negative equilibrium concentrations of free NRF, free sMAF, NRF–sMAF heterodimer, and sMAF–sMAF homodimer at each pair of total NRF and sMAF concentrations.

ARE was treated as trace and non-depleting. Fractional occupancy of ARE by NRF–sMAF was calculated as:

$$f_{\mathrm{NRF-sMAF-ARE}} = \frac{[\mathrm{NRF-sMAF}]/K_{d,\mathrm{NRF-sMAF-ARE}}}{1 + [\mathrm{NRF-sMAF}]/K_{d,\mathrm{NRF-sMAF-ARE}} + [\mathrm{sMAF-sMAF}]/K_{d,\mathrm{sMAF-sMAF-AR}}}$$

The corresponding sMAF–sMAF-bound and free ARE fractions were calculated by normalization of the same three occupancy terms. The same equilibrium calculation was applied to each set of experimentally determined apparent K_d values without additional parameter fitting. To retain the general sMAF framework, the concentration axis was treated as a representative total sMAF concentration parameterized using the MAFG interaction set. In a complete mixed-sMAF system, the contributions of MAFF, MAFG, and MAFK would require reaction-specific weighting according to the full apparent K_d matrix and therefore cannot be reduced exactly to a single universal concentration scale. Because endogenous sMAF composition and concentration ranges were not specified, the present calculation used the experimentally determined MAFG parameter set and numerically equated the representative total sMAF concentration with total MAFG concentration. Accordingly, the calculated concentration axis represents a MAFG-parameterized representative total sMAF concentration rather than measured total cellular sMAF abundance.

Calculation of sMAF concentration at maximal NRF–sMAF–ARE occupancy

For Fig. S8, the coupled-equilibrium occupancy relationship described above was evaluated at fixed total NRF concentrations of 0.1, 1, 10, or 100 nM. For each ARE sequence, the sMAF concentration corresponding to maximal NRF–sMAF–ARE occupancy was determined analytically. Let $x = [\mathrm{sMAF}]_{\mathrm{free}}$. The occupancy maximum was defined by

$$\frac{df_{\mathrm{NRF-sMAF-ARE}}}{dx} = 0,$$

which reduces to

$$2x_{max}^{3} + K_{d,NRF-sMA}\ x_{max}^{2} - K_{d,NRF-s}\quad K_{d,sMAF-sM}\quad K_{d,sMAF-sMAF-ARE} = 0$$

The unique positive real root, x_{max}, was converted to total sMAF concentration using the sMAF mass-balance relationship defined above, and the corresponding NRF–sMAF–ARE occupancy was calculated from the coupled-equilibrium occupancy equation. The resulting total sMAF concentration at maximal occupancy and the corresponding occupancy were plotted as the x- and y-coordinates, respectively. For Fig. S8, MAFG was used to represent the total sMAF concentration in the coupled-equilibrium calculation.

Calculation of finite-time occupancy variability

A simplified two-state binding system was used to examine how residence time affects finite-time occupancy variability at fixed equilibrium occupancy. All targets were assigned an equilibrium occupancy of 0.5 and an observation window of 1 h. Residence half-life ($t_{1/2}$) was varied from 1 to 10,000 s. For each residence half-life, the dissociation rate constant was calculated as:

$$k_{\text{off}} = \frac{\ln(2)}{t_{1/2}}$$

The occupancy correlation time was calculated as:

$$\tau_c = \frac{(1-p)t_{1/2}}{\ln(2)}$$

where p is the equilibrium occupancy. The variance of time-averaged occupancy over an observation window T was calculated as:

$$\text{Var}(\bar{p}) = \frac{2p(1-p)}{T^2}\left[T\tau_c - \tau_c^2\left(1 - e^{-T/\tau_c}\right)\right]$$

The coefficient of variation of time-averaged occupancy was then calculated as:

$$\text{CV} = \frac{\sqrt{\text{Var}(\bar{p})}}{p}$$

The experimentally determined WT NRF2–MAFG–ARE residence half-life of 16.15 s was included as a reference point.

Cellular ARE-reporter assay

HEK-293 cells were transiently transfected with 20 ng of a firefly luciferase reporter containing the ARE sequence CCTGAGTCACG, 80 ng of pCI-NRF2-K7, and increasing amounts of pCI-MAFG (0, 5, 10, 20, 40, or 80 ng) per well. Empty pCI vector was added to maintain a constant total of 220 ng plasmid DNA per well across all conditions. The empty-vector control received the ARE reporter and empty pCI vector without NRF2-K7 or MAFG.

For each transfection, plasmid DNA was mixed with 0.20 μL P3000 reagent in Opti-MEM to a final volume of 5 μL. Separately, 0.225 μL Lipofectamine 3000 was diluted in Opti-MEM to 5 μL. The two mixtures were combined and incubated for 10 min at room temperature, and 10 μL was added to each well. Firefly luciferase activity was measured 16 h after transfection and normalized to the mean activity of the empty-vector control.

Each experiment included four independently cultured and transfected wells per condition, and the experiment was independently repeated three times with similar results. Data shown in Fig. 6G represent the mean ± SD of four wells from one representative experiment.

Statistical comparisons were performed using two-tailed unpaired Student's t tests as indicated. A quadratic contrast was evaluated within a one-way ANOVA across the six ordered MAFG dose groups (0, 5, 10, 20, 40, and 80 ng), treating the groups as equally spaced ordered factor levels. The empty-vector control was excluded from the trend analysis. Statistical significance was defined as $P < 0.05$.

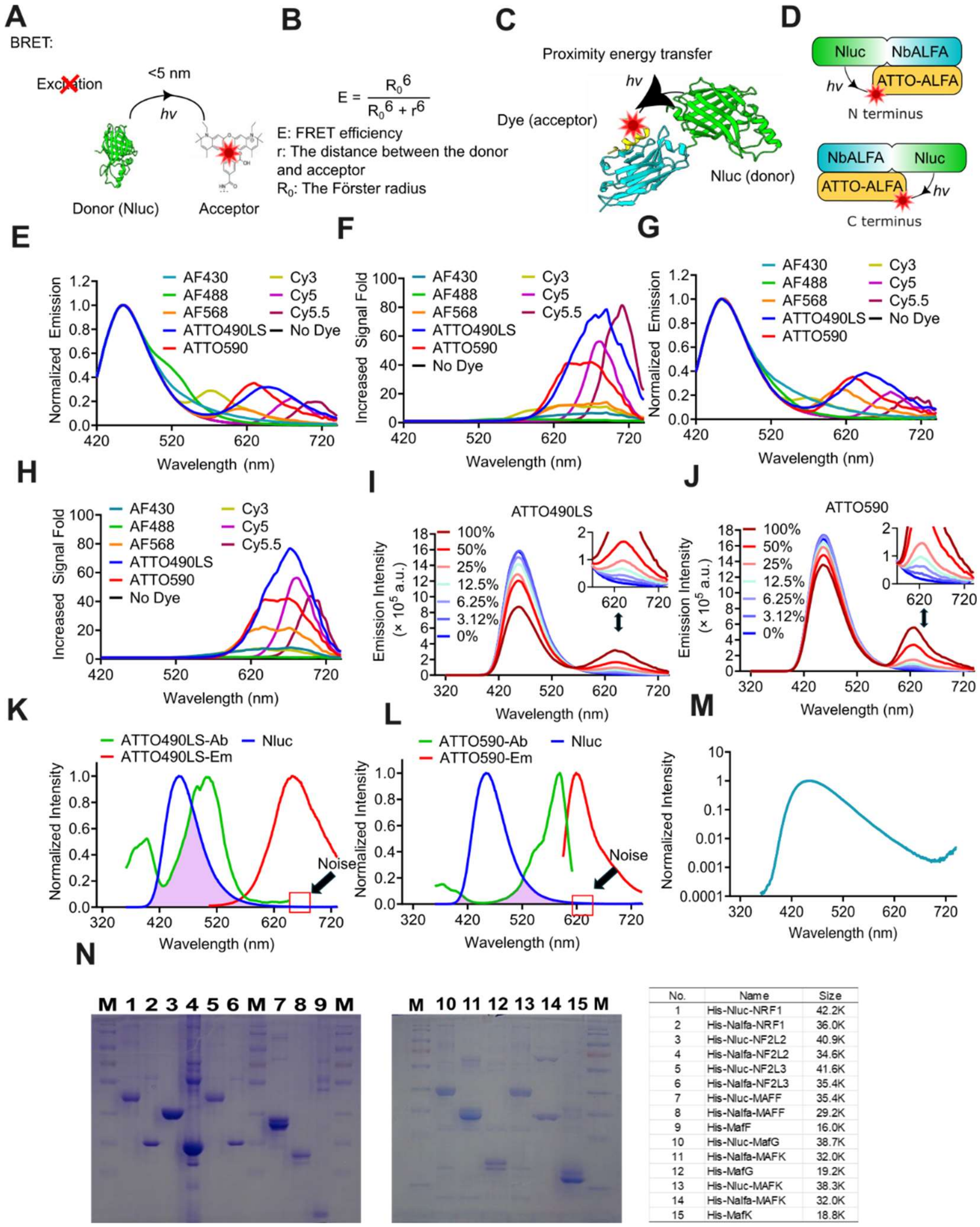

No.	Name	Size
1	His-Nluc-NRF1	42.2K
2	His-Nalfa-NRF1	36.0K
3	His-Nluc-NF2L2	40.9K
4	His-Nalfa-NF2L2	34.6K
5	His-Nluc-NF2L3	41.6K
6	His-Nalfa-NF2L3	35.4K
7	His-Nluc-MAFF	35.4K
8	His-Nalfa-MAFF	29.2K
9	His-MafF	16.0K
10	His-Nluc-MafG	38.7K
11	His-Nalfa-MAFK	32.0K
12	His-MafG	19.2K
13	His-Nluc-MAFK	38.3K
14	His-Nalfa-MAFK	32.0K
15	His-MafK	18.8K

Figure S1. Supporting characterization of the NanoBRET measurement framework. (A) Representative NanoBRET donor–acceptor configuration, illustrating proximity-dependent energy transfer from a NanoLuc (Nluc) donor to a fluorophore acceptor without external excitation. **(B)** Förster resonance energy-transfer efficiency relationship, illustrating the strong distance dependence of donor–acceptor energy transfer. **(C, D)** Structural and schematic representations of the NbALFA–ALFA reference system used to characterize the NanoBRET readout. Nluc-tagged NbALFA serves as the donor and binds the fluorophore-labeled ALFA

peptide acceptor, providing a defined high-affinity donor–acceptor interaction for assay evaluation. **(E–H)** NanoBRET emission profiles for ALFA peptides labeled at the C terminus **(E, F)** or N terminus **(G, H)** with the indicated fluorophores under saturating NbALFA–ALFA binding conditions. Both labeling orientations supported proximity-dependent energy transfer. N-terminal labeling provided consistently strong signal and was therefore selected as the standard ALFA acceptor configuration for subsequent protein–protein NanoBRET measurements. **(I, J)** Luminescence emission spectra of the selected N-terminally labeled ALFA–ATTO490LS **(I)** and ALFA–ATTO590 **(J)** acceptors across defined donor–acceptor engagement fractions generated by premixing labeled and unlabeled ALFA peptides. Increasing labeled-acceptor engagement produced a progressive decrease in Nluc donor emission and a corresponding increase in acceptor emission, directly linking the spectral response to the fraction of engaged donor–acceptor pairs. **(K, L)** Spectral characteristics of the Nluc–ATTO490LS **(K)** and Nluc–ATTO590 **(L)** donor–acceptor pairs in the selected N-terminal ALFA acceptor configuration. Nluc donor emission and acceptor excitation and emission profiles are shown to illustrate the spectral relationships underlying energy transfer and the residual donor emission contributing to acceptor-channel background. **(M)** Log-scaled Nluc emission spectrum illustrating the progressive decrease in donor emission at longer wavelengths and the contribution of residual donor emission to acceptor-channel background in the selected NanoBRET configuration. **(N)** SDS–PAGE analysis of purified protein constructs used in subsequent NanoBRET measurements. Proteins were visualized by Coomassie Brilliant Blue staining; M denotes the molecular-weight marker. Together, these analyses established the N-terminal ALFA acceptor configuration for subsequent protein–protein NanoBRET measurements and, using the picomolar-affinity NbALFA–ALFA reference pair under near-saturating conditions, generated defined donor–acceptor engagement fractions largely decoupled from changes in binding equilibrium, enabling direct quantitative characterization of the NanoBRET signal response.

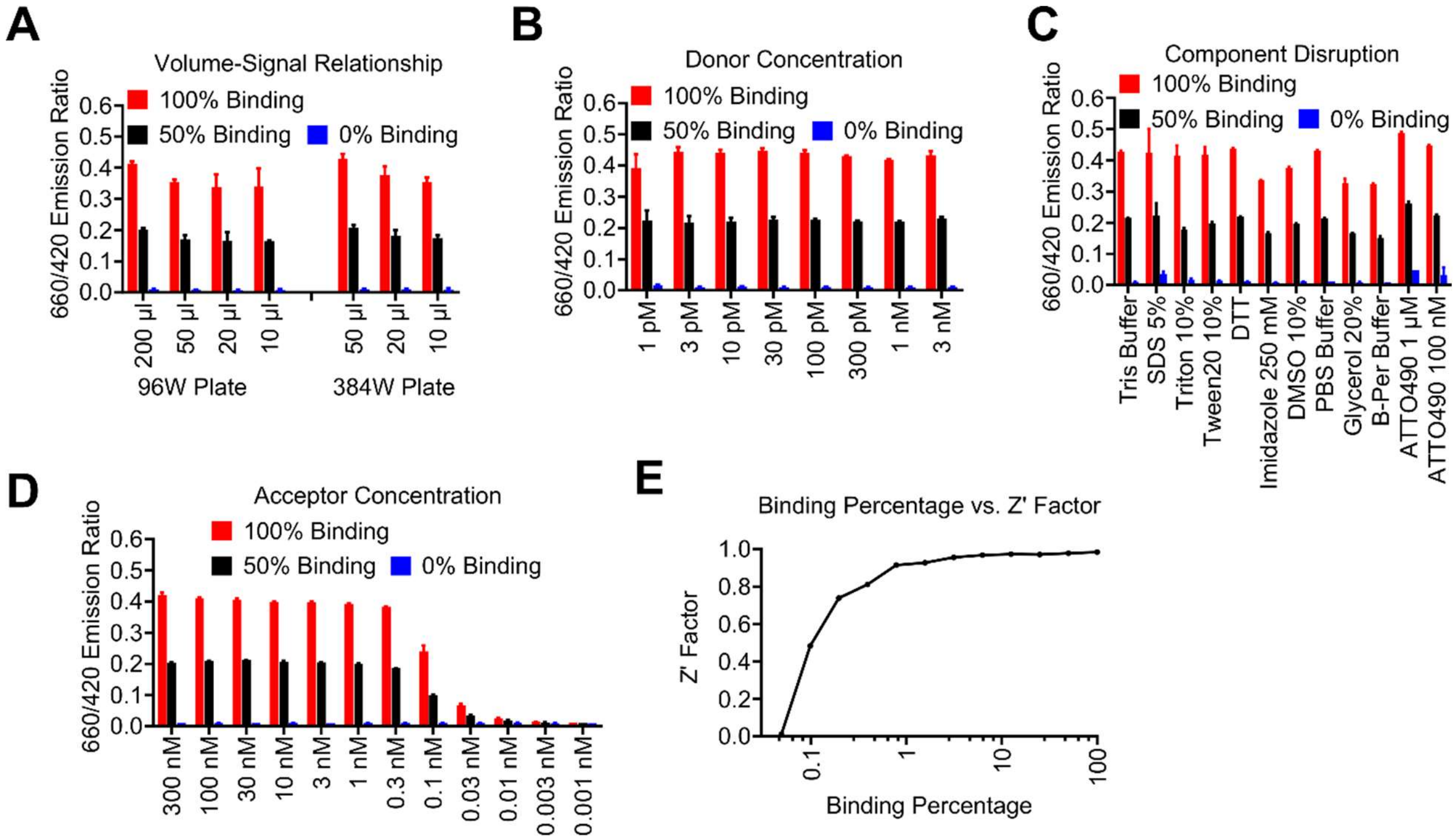

Figure S2. Analytical robustness of the NanoBRET readout across defined and equilibrium-dependent engagement readouts. (A) NanoBRET emission ratios measured across assay volumes from 10 to 200 µL in 96-well plates and from 10 to 50 µL in 384-well plates using defined 0%, 50%, and 100% donor–acceptor engagement fractions. The ratiometric NanoBRET response remained comparable across plate formats and assay volumes. **(B)** Effect of Nluc donor concentration over a 3,000-fold range on the NanoBRET emission ratio. Defined engagement fractions remained distinguishable across the tested donor concentrations, indicating that the ratiometric readout is largely insensitive to changes in absolute donor abundance. **(C)** NanoBRET emission ratios measured in the presence of high concentrations of representative buffers, detergents, reducing agents, solvents, purification components, and free fluorophore. The defined engagement readouts remained broadly preserved across these conditions, supporting resistance of the ratiometric readout to changes in assay composition. **(D)** Effect of N-terminally labeled ALFA–ATTO490LS acceptor concentration on the NanoBRET signal. At acceptor concentrations well above the picomolar NbALFA–ALFA dissociation constant, the predefined 0%, 50%, and 100% labeled-engagement conditions produced stable and clearly separated NanoBRET signals. As acceptor concentration approached and fell below the binding-equilibrium range, incomplete NbALFA–ALFA engagement progressively reduced the overall signal, reflecting concentration-dependent redistribution between unengaged and engaged fractions. Importantly, the 0%, 50%, and 100% labeled conditions remained clearly ordered and distinguishable at the same acceptor concentrations, consistent with the engagement-dependent NanoBRET response established in Fig. 1D. Thus, NanoBRET continued to resolve differences in labeled donor–acceptor engagement while simultaneously reporting changes in the underlying equilibrium-dependent bound fraction. **(E)** Z′ factor calculated across the defined donor–acceptor engagement fractions used in Fig. 1C and D, with the 0% engagement condition serving as the negative reference.

This analysis extends the engagement-fraction measurements shown in the main figure by quantifying the precision with which incremental differences in pair engagement can be resolved across the measured range. Together, the defined engagement-fraction measurements in Fig. 1C and D and the extended analyses shown here establish the quantitative basis for subsequent NanoBRET measurements: fractional donor–acceptor engagement produces a proportional normalized mBRET response, and differences in engagement can be resolved with high precision. The additional robustness and acceptor-concentration analyses further show that this quantitative relationship is maintained across diverse measurement conditions and remains informative when pair engagement is coupled to a concentration-dependent binding equilibrium.

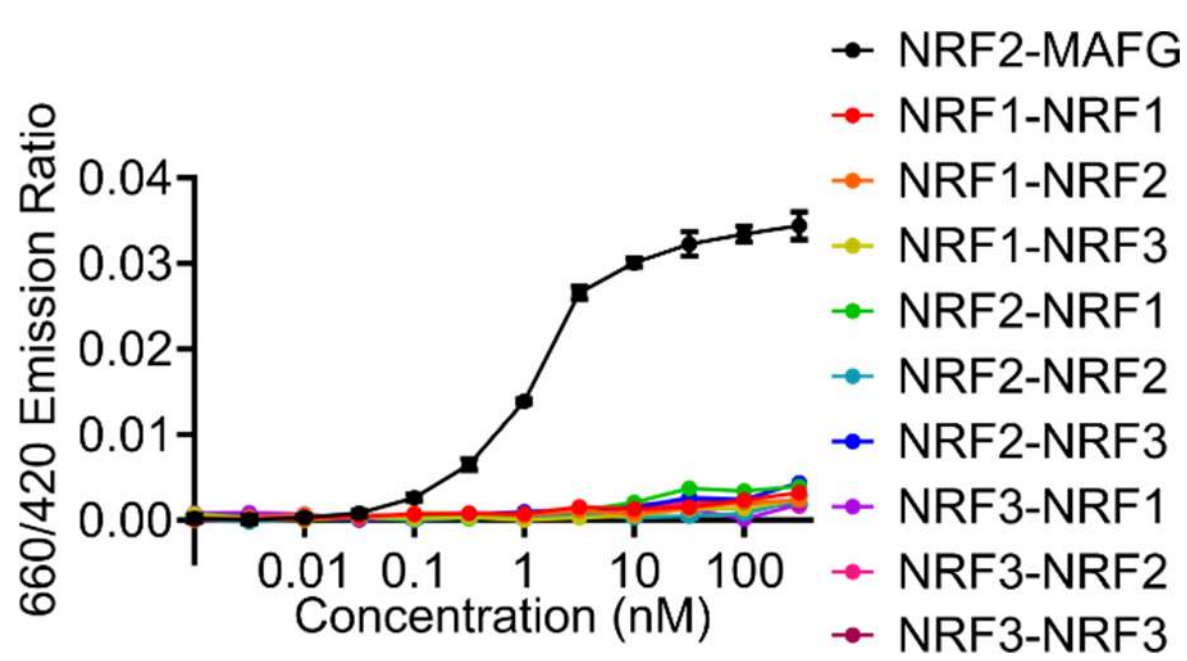

Figure S3. Complete NRF–NRF interaction matrix shows no detectable homo- or heterotypic NRF dimerization under the measured conditions. Pairwise NanoBRET titrations were performed for all donor–acceptor combinations among NRF1, NRF2, and NRF3. All nine NRF–NRF homo- and heterotypic combinations remained at baseline across the tested concentration range, with no measurable engagement detected up to 316 nM, whereas NRF2–MAFG produced a saturable NanoBRET response and is shown as a representative positive interaction control. These measurements extend the representative NRF2–NRF2 result shown in Fig. 2D and demonstrate that the absence of detectable NRF–NRF pairing is shared across the NRF1–3 family under the measured conditions. Together with the full NRF/sMAF interaction landscape in Fig. 2, these data constrain the measurable dimer pool to NRF–sMAF and sMAF–sMAF combinations and define the accessible dimers considered in subsequent ARE-engagement and occupancy analyses.

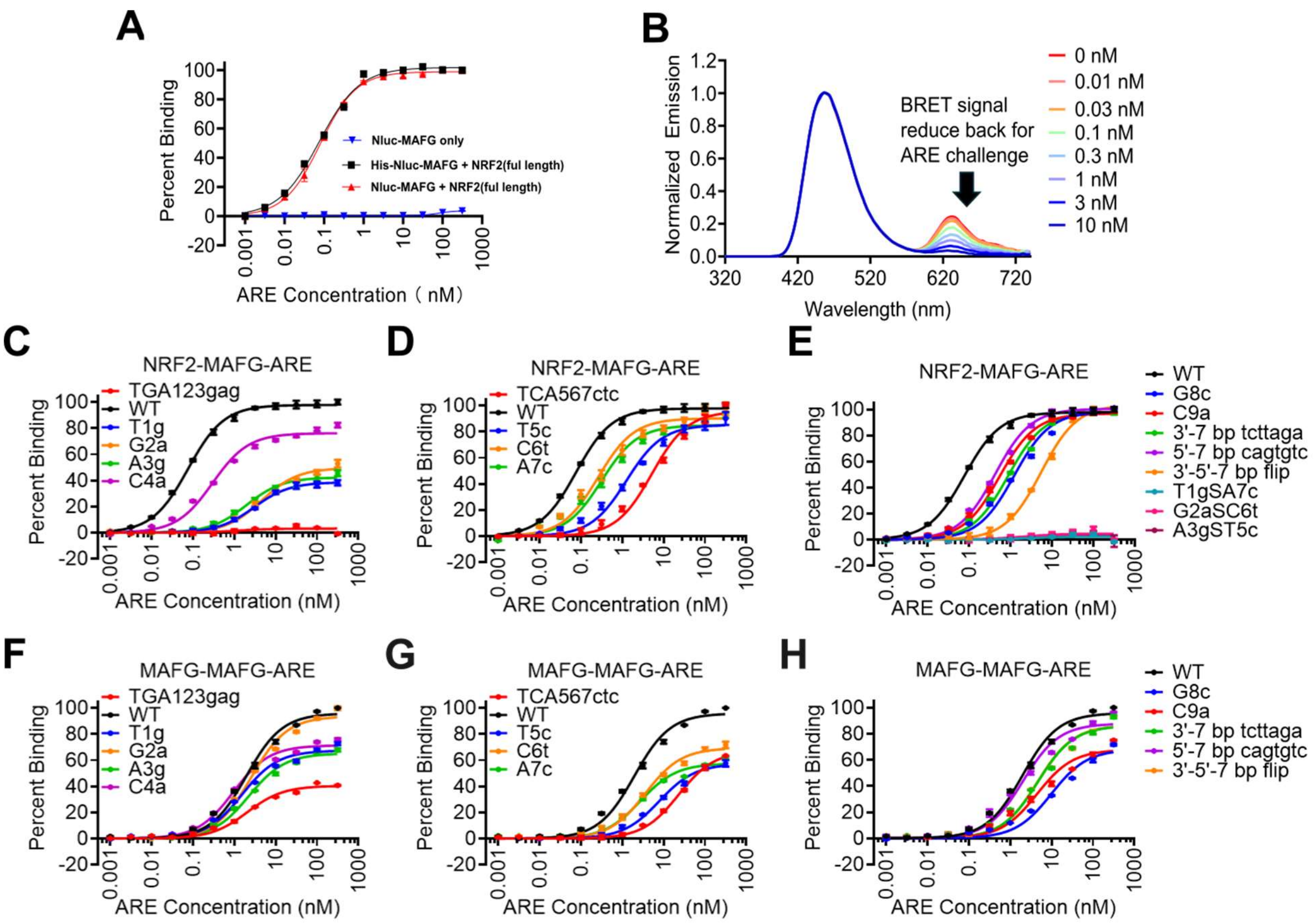

Figure S4. Validation of protein–DNA NanoBRET and extended sequence-dependent profiling of NRF2–MAFG and MAFG–MAFG ARE engagement.

(A) ARE-binding titrations using a reverse-labeling configuration, in which Nluc was fused to MAFG and full-length NRF2 remained unlabeled, to account for the position sensitivity of the NanoBRET signal and assess whether the isolated NRF2 Neh1 domain recapitulates the DNA-binding behavior of full-length NRF2. Full-length NRF2 combined with either His-Nluc-MAFG or Nluc-MAFG produced comparable saturable binding profiles, whereas Nluc-MAFG alone generated minimal signal. These results indicate that the His tag did not appreciably affect ARE engagement and support the use of Neh1 as a representative DNA-binding module of NRF2 in the primary assay configuration. **(B)** Competition of ATTO590-labeled ARE engagement by increasing concentrations of unlabeled ARE. Increasing unlabeled ARE reduced acceptor emission and restored donor emission, consistent with displacement of the labeled ARE probe from the measured protein–DNA complex. This competition behavior confirms that the NanoBRET response used for the ARE sequence comparisons reflects specific engagement of the labeled ARE probe. Together, these extended titration series support the dimer- and sequence-dependent apparent affinity landscape summarized in Fig. 3, while competition with unlabeled ARE independently validates assignment of the protein–DNA NanoBRET signal to ARE engagement. **(C–E)** Extended ARE-binding titrations for the NRF2–MAFG–ARE complex measured using Nluc-tagged ΔNRF2 in the presence of excess unlabeled MAFG. **(C)** Binding profiles for WT ARE and variants affecting the 5′ core region, including individual substitutions at positions 1–4 and the combined position 1–3 mutant. **(D)** Binding profiles for variants affecting

positions 5–7, including individual substitutions and the combined position 5–7 mutant. **(E)** Binding profiles for additional ARE variants affecting positions 8 and 9, symmetric paired positions across the ARE core, and the 5′ or 3′ flanking regions. **(F–H)** Corresponding extended ARE-binding titrations for the MAFG–MAFG–ARE complex, organized according to the same sequence-variant groups shown in **(C–E)**. Binding responses in **(C–H)** were normalized to WT NQO1 ARE and extend the sequence-dependent engagement profiles presented in Fig. 3, revealing distinct effects of ARE variation on NRF2–MAFG and MAFG–MAFG recognition.

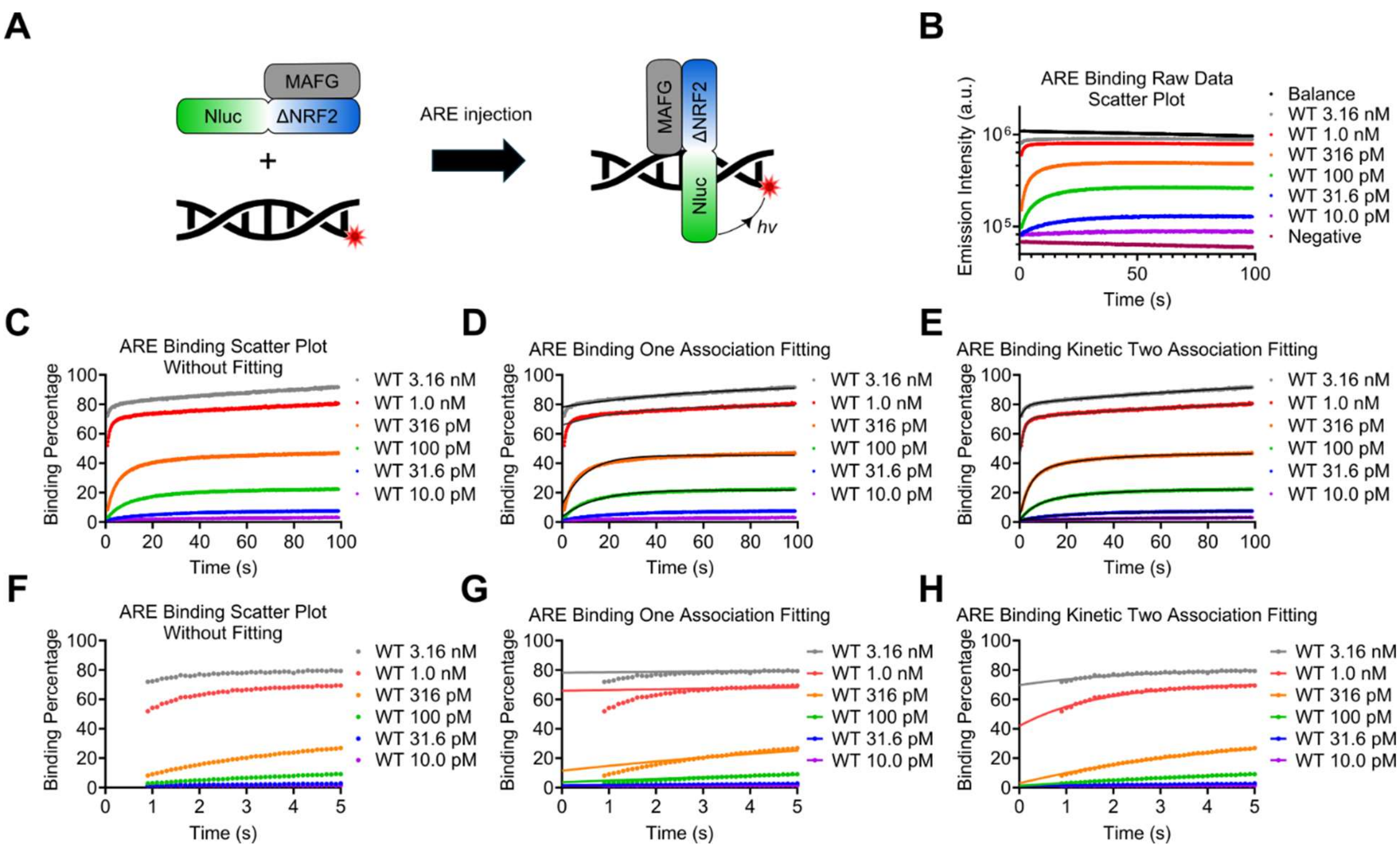

Figure S5. Biphasic association kinetics provide evidence for coupling within NRF2–MAFG–ARE assembly. (A) Schematic of the injection-based NanoBRET kinetic assay used to monitor NRF2–ARE engagement during NRF2–MAFG–ARE assembly. NanoLuc-tagged NRF2 was preassembled with unlabeled MAFG, and association was initiated by injection of ATTO590-labeled ARE. **(B)** Representative raw acceptor-channel signals for the WT ARE concentration series. Pre-equilibrated balanced and negative reference conditions are shown for kinetic normalization. The approximately parallel signal behavior of these reference conditions on a logarithmic scale reflects the stable proportional relationship between the balanced and negative signals over the measurement window. **(C)** Normalized WT ARE association traces shown as raw scatter-point data without fitted curves. Each trace contains 982 individually acquired data points without interpolation. **(D)** The same association traces described using a single-exponential association model, representing a simple single-phase association process. Systematic deviations are apparent during the early association period, particularly at higher ARE concentrations. **(E)** Empirical two-phase association analysis of the same traces resolves a

rapid initial component together with a slower secondary component. **(F–H)** Expanded views of the first 5 s corresponding to panels C–E, respectively. The early-time data directly resolve the rapid signal increase and highlight the reproducible deviation from simple single-phase association, whereas the two-phase description captures both the rapid and slower components of the measured NRF2–ARE engagement behavior. Panels A and H are also shown in Fig. 4A and B, respectively, to present the assay configuration and the key early kinetic behavior in the main-text analysis. Together, these data show that NRF2–ARE engagement during NRF2–MAFG–ARE formation exhibits reproducible biphasic kinetics that are not adequately described as a simple isolated one-step DNA-association reaction. The resolved fast and slow components provide kinetic evidence that the measured engagement remains coupled to an additional assembly-associated process within the NRF2–MAFG–ARE network.

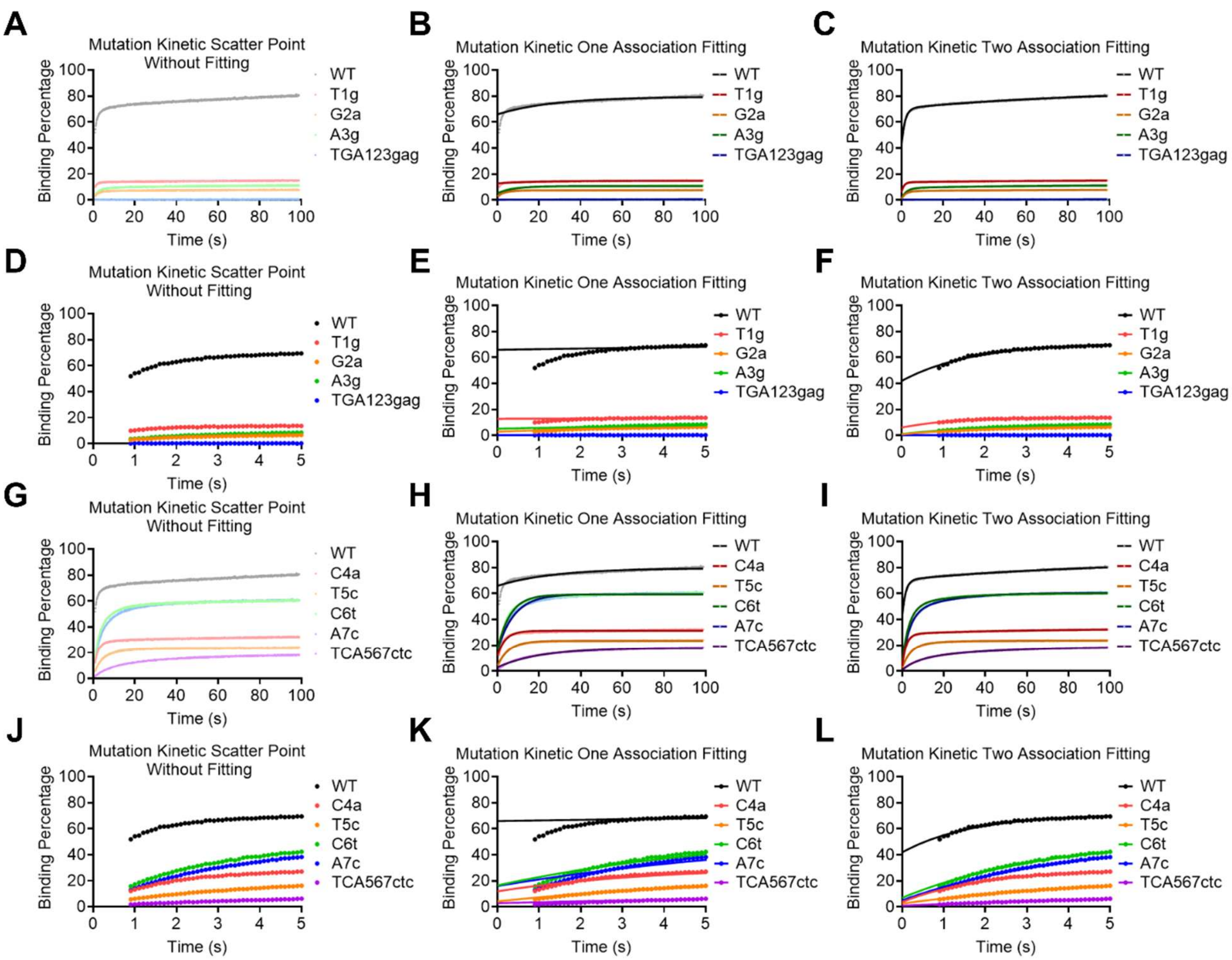

Figure S6. Extended association kinetics of WT and mutant ARE sequences. (A–C) Full 100-s association kinetics for WT ARE and position 1–3 mutant probes, including the single-position variants T1g, G2a, and A3g and the combined TGA123gag mutant. **(A)** Raw association traces shown without fitted curves. **(B)** Single-exponential descriptions of the measured kinetics. **(C)** Two-phase association analysis resolving fast and slower kinetic components. **(D–F)** Expanded views of the first 5 s corresponding to panels A–C, respectively,

highlighting the early association behavior of WT and position 1–3 ARE variants. Mutations at positions 1–3 markedly reduced the measured engagement signal, with the combined TGA123gag mutant approaching the limit of detectable binding. **(G–I)** Full 100-s association kinetics for WT ARE and position 4–7 mutant probes, including C4a, T5c, C6t, A7c, and the combined TCA567ctc mutant. **(G)** Raw association traces shown without fitted curves. **(H)** Two-phase association analysis. **(I)** Single-exponential descriptions of the same traces. **(J–L)** Expanded views of the first 5 s corresponding to panels G–I, respectively. Several position 4–7 variants retained substantial engagement but displayed altered association amplitudes and kinetic behavior relative to WT. Fitted curves are shown as thin black lines overlaid on the raw data points; in traces with close agreement, fitted curves may be visually obscured by the underlying data. Together, these extended kinetic series show that the multiphasic association behavior identified for WT ARE is retained across measurable ARE sequence variants, while sequence changes differentially alter the kinetics of NRF2–MAFG–ARE engagement. These data support the sequence-dependent kinetic comparisons summarized in Fig. 4.

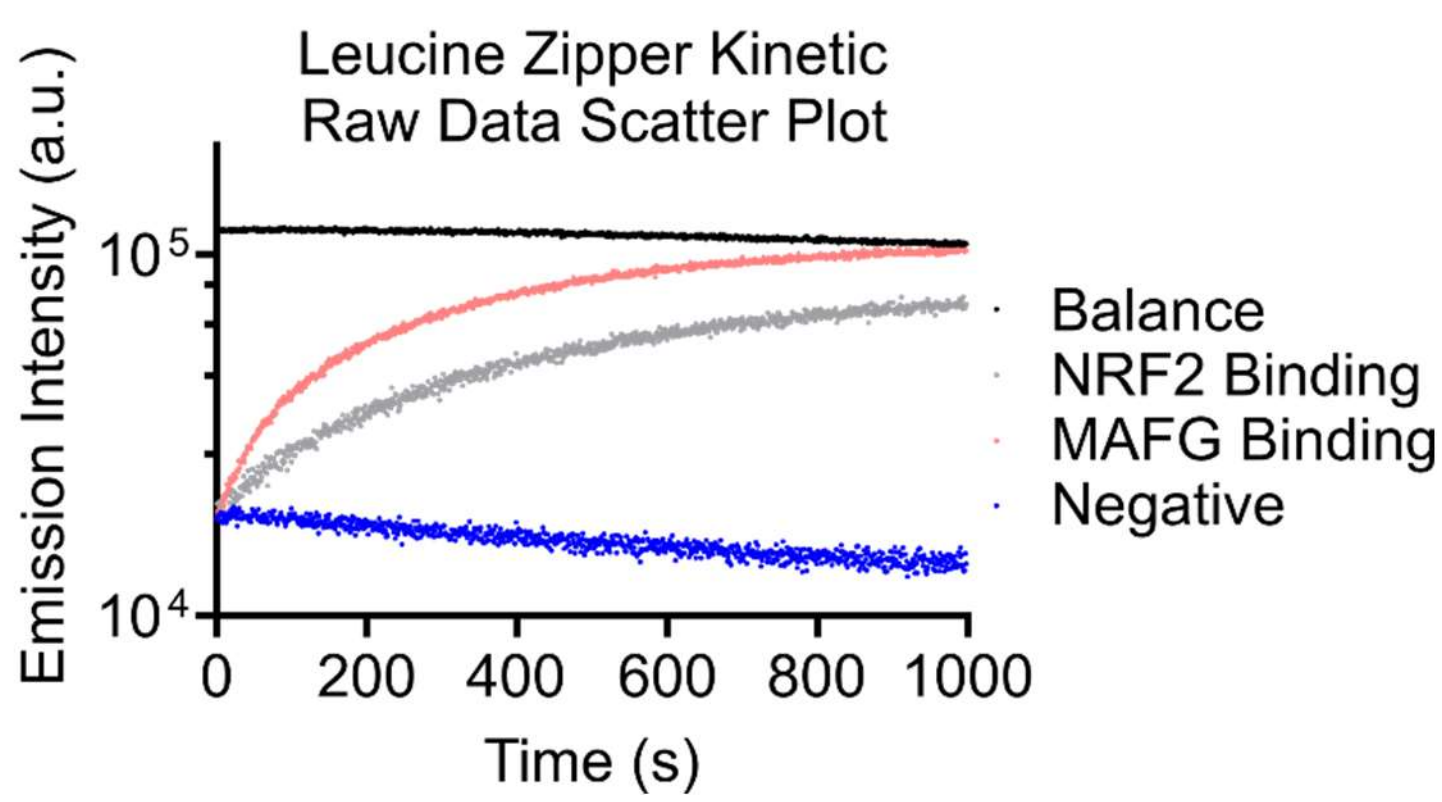

Figure S7. Raw NanoBRET reference signals for alternative NRF2–MAFG–ARE assembly-order measurements. Representative raw acceptor-channel signals recorded under the alternative component-addition conditions used for the assembly-order experiments in Fig. 5. Pre-equilibrated balanced and negative reference conditions corresponding to these assay configurations are shown. The approximately parallel behavior of the reference traces on a logarithmic scale reflects a stable proportional relationship over the measurement window and supports normalization of the route-dependent kinetic measurements presented in Fig. 5.

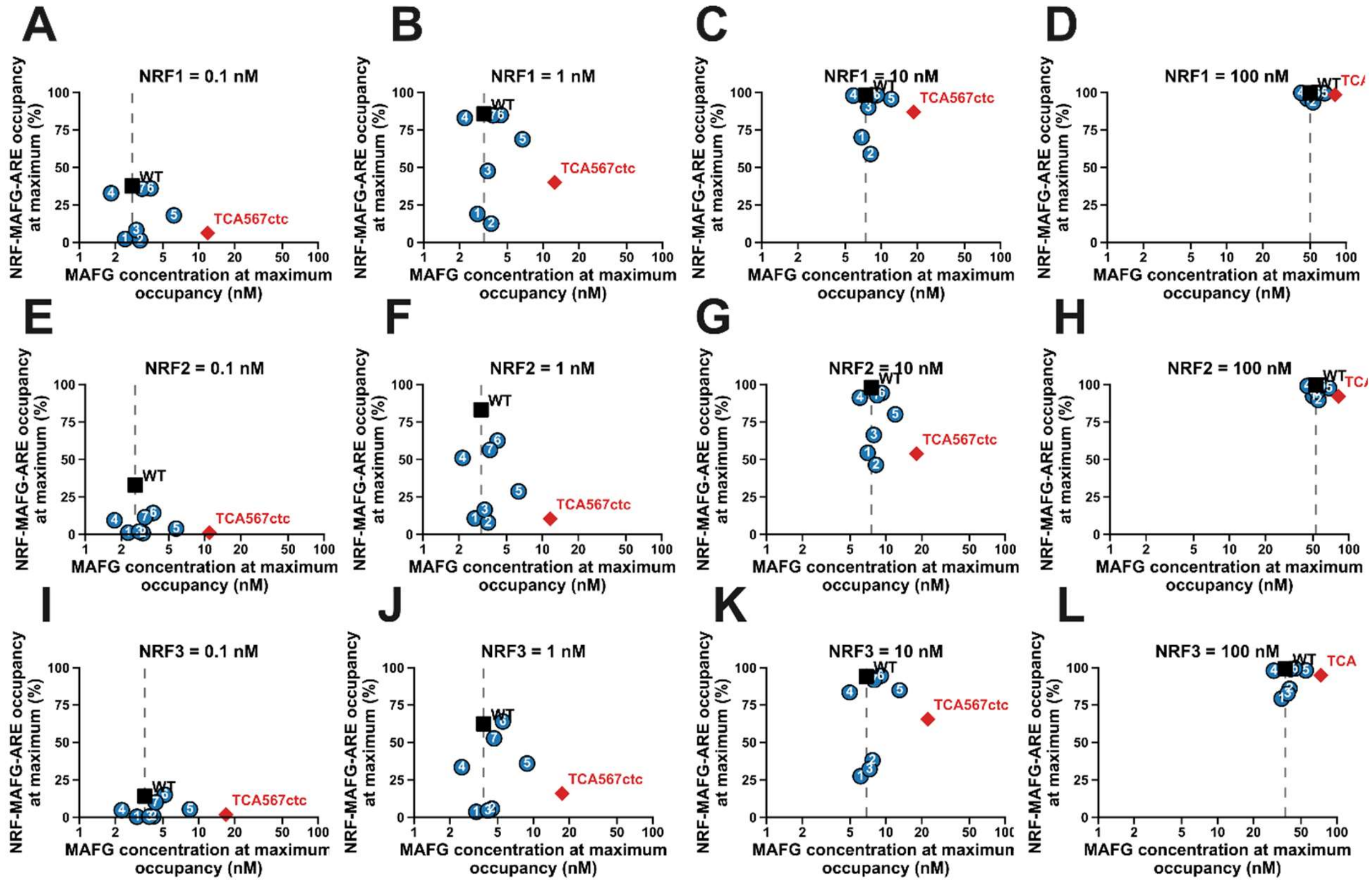

Figure S8. Model-predicted ARE sequence-dependent sensitivity to MAFG-mediated constraint of NRF–MAFG–ARE occupancy. (A–D) NRF1, **(E–H)** NRF2, and **(I–L)** NRF3 were evaluated at fixed total NRF concentrations of 0.1, 1, 10, and 100 nM, respectively, from left to right. For each ARE sequence, the coupled-equilibrium calculation was used to determine the total MAFG concentration at which NRF–MAFG–ARE occupancy reached its maximum and the corresponding occupancy at that NRF concentration. Numerals 1–7 denote the single-base ARE variants T1g, G2a, A3g, C4a, T5c, C6t, and A7c, respectively. WT and TCA567ctc are shown separately, and the vertical dashed line marks the WT MAFG concentration at maximal occupancy in each panel. As illustrated by the biphasic MAFG response in Fig. 6D, the occupancy maximum marks the MAFG concentration beyond which further MAFG increasingly favors the competing MAFG–MAFG complex and reduces NRF–MAFG–ARE occupancy. A left-shifted maximum therefore indicates greater sensitivity to MAFG-dependent constraint and earlier loss of NRF–MAFG–ARE occupancy as MAFG increases. In contrast, a right-shifted maximum indicates greater resistance to this constraint and retention of NRF–MAFG–ARE occupancy over a broader MAFG concentration range. The sequence-dependent distribution of these turning points illustrates concentration-dependent sequence filtering within the parameterized occupancy framework, in which increasing MAFG preferentially constrains MAFG-sensitive sequences while preserving NRF–MAFG–ARE occupancy at less MAFG-sensitive sequences. Changes in occupancy across the NRF concentration series further illustrate that a fixed ARE can remain

responsive to increasing NRF abundance while retaining its distinct MAFG-dependent response profile.

Supporting Tables

Fitted parameters are reported as best-fit estimates, with the uncertainty measure defined in the relevant table. Exact confidence limits and complete fitting outputs are provided in the accompanying Supporting Information Excel file, which serves as the primary source for detailed fit statistics.

Table S1. Spectral parameters of donor–acceptor pairs used for NanoBRET evaluation.

Name	Excitation (nm)	Emission (nm)	Stokes Shift (nm)	EC_{max} ($\times 10^{4}$ $M^{-1}cm^{-1}$)	QY	J(λ) ($\times 10^{14}$ $M^{-1}cm^{-1}nm^{4}$)	R_0 (nm)
AF430	442	551	109	1.60	0.23	3.59	2.88
AF488	486	517	31	7.30	0.92	15.0	4.60
AF568	570	603	33	8.80	0.69	5.21	3.68
ATTO490ls	502	653	151	4.00	0.30	11.4	3.64
ATTO590	589	619	30	1.20	0.80	5.56	3.81
Cy3	548	566	18	1.36	0.15	15.2	3.41
Cy5	647	669	22	25.0	0.30	3.60	3.01
Cy5.5	673	698	25	25.0	0.28	2.82	2.85

The excitation and emission maxima were determined from the measured excitation and emission spectra, respectively. Dye NHS ester stocks were diluted to 10 μM in 10 mM Tris buffer (pH 7.4) and incubated for 2 h at 23°C, after which the samples were further diluted to 10 nM for spectral measurements. Reported maximum molar extinction coefficients (EC_{max}) and fluorescence quantum yields (QY) were obtained from FPbase. The spectral overlap integral, J(λ), and Förster radius, R0, were calculated from the donor emission and acceptor absorption spectra using the equations below.

$$J(\lambda) = \int F_D(\lambda)\, \varepsilon_A(\lambda)\, \lambda^4\, d\lambda$$

$$R_0 = 0.02108(\kappa^2\, Q_D\, n^{-4}\, J(\lambda))^{1/6}$$

The parameters used for Nluc were Q_D: 0.45, n: 1.4, κ^2: 2/3.

Table S2. Comprehensive quantification of NRF-MAF interaction affinities by NanoBRET.

Donor	Acceptor	K_d (nM)	95% CI	R^2	B_{max}
NRF1	MAFF	3.402	±0.73	0.9958	22.99±0.96
NRF1	MAFG	1.472	±0.387	0.9925	37.86±1.9
NRF1	MAFK	1.328	±0.481	0.9857	36.78±2.54
NRF2	MAFF	15.850	±3.575	0.9955	22.43±1.235
NRF2	MAFG	1.254	±0.208	0.9972	34.18±1.04
NRF2	MAFK	1.498	±0.13	0.9993	34.87±0.555
NRF3	MAFF	40.140	±19.715	0.9834	10.08±1.418
NRF3	MAFG	3.062	±0.383	0.9984	14.52±0.365
NRF3	MAFK	29.330	±6.14	0.9962	13.43±0.78
MAFF	MAFF	3.263	±2.424	0.9634	16.24±2.035
MAFF	MAFG	4.591	±3.194	0.9684	15.48±1.92
MAFF	MAFK	27.770	±11.725	0.9855	18.05±2.03
MAFF	NRF1	3.568	±0.707	0.9966	54.73±2.1
MAFF	NRF2	14.860	±3.27	0.9955	47.87±2.565
MAFF	NRF3	38.270	±17.115	0.9859	43.67±5.54
MAFG	MAFF	12.930	±2.065	0.9975	31.92±1.23
MAFG	MAFG	2.450	±0.691	0.9919	33.24±1.845
MAFG	MAFK	7.235	±2.231	0.9901	32.32±2.205
MAFG	NRF1	1.403	±0.393	0.9914	94.07±5.02
MAFG	NRF2	1.312	±0.242	0.9965	84.3±2.86
MAFG	NRF3	15.470	±4.515	0.9916	48.17±3.505
MAFK	MAFF	17.320	±3.615	0.9956	33.03±1.775
MAFK	MAFG	2.734	±0.634	0.9943	31.01±1.445
MAFK	MAFK	16.160	±3.455	0.9957	26.68±1.415
MAFK	NRF1	1.392	±0.353	0.9931	90.15±4.345
MAFK	NRF2	1.535	±0.287	0.9967	83.75±2.84
MAFK	NRF3	29.500	±3.765	0.9985	29.11±1.055

Equilibrium dissociation constants (K_d) were obtained by nonlinear regression to a single-site binding function. Values are best-fit estimates, and the reported ± values provide compact summaries of the corresponding 95% profile-likelihood confidence intervals; exact lower and upper limits and complete fit outputs are provided in Supporting Information Excel Sheet 2. R^2 indicates overall goodness of fit. Reciprocal donor–acceptor configurations produced comparable K_d values.

Table S3. Thermodynamic parameters of NRF–MAFG–ARE and MAFG–MAFG–ARE interactions measured by NanoBRET.

A: NRF1-MAFG-ARE interaction

Acceptor	Kd (nM)	95% CI (nM)	R²	Bmax ± 95% CI (%)
**WT**	0.057	±0.021	0.978	93.43 ± 5.46
**TGA123gac***	N.A.	-	0.565	-0.805 ± 1.945
**TCA567ctc**	0.871	±0.215	0.992	88.51 ± 4.19
**T1g**	1.384	±0.558	0.975	41.19 ± 3.55
**G2a**	2.581	±1.055	0.974	49.75 ± 4.7
**A3g**	0.394	±0.13	0.985	42.76 ± 2.57
**C4a**	0.058	±0.019	0.982	72.4 ± 3.84
**T5c**	0.217	±0.068	0.986	79.38 ± 4.25
**C6t**	0.072	±0.027	0.978	83.69 ± 5.06
**A7c**	0.068	±0.035	0.947	71.84 ± 6.85

B: NRF2-MAFG-ARE interaction

Acceptor	Kd (nM)	95% CI (nM)	R²	Bmax ± 95% CI (%)
**WT**	0.076	±0.008	0.999	97.77 ± 1.46
**TGA123gac***	N.A.	-	0.494	3.194 ± 1.76
**TCA567ctc**	5.221	±1.573	0.990	95.59 ± 6.58
**T1g**	2.969	±0.383	0.998	38.69 ± 1.05
**G2a**	4.647	±1.406	0.990	49.52 ± 3.41
**A3g**	1.975	±0.534	0.992	42.32 ± 2.35
**C4a**	0.299	±0.089	0.989	76.15 ± 3.95
**T5c**	1.266	±0.38	0.990	85.21 ± 4.97
**C6t**	0.261	±0.096	0.982	90.15 ± 5.82
**A7c**	0.316	±0.118	0.982	84.9 ± 5.64
**G8c**	1.271	±0.447	0.994	92.65 ± 8.11
**C9a**	0.559	±0.118	0.998	93.13 ± 4.43
**3'-7 bp tcttaga**	0.96	±0.281	0.996	94.82 ± 6.61
**5'-7 bp cagtgtc**	0.446	±0.072	0.999	98.62 ± 3.51
**3'-5'-7 bp flip**	6.365	±0.92	0.999	106.3 ± 4.8
**T1gSA7c**	20.67	±11.22	0.966	9.518 ± 2.53
**G2aSC6t**	16.38	±10.53	0.955	12.81 ± 3.793
**A3gST5c**	20.49	±11.8	0.958	11.05 ± 3.172

C: NRF3-MAFG-ARE interaction

Acceptor	Kd (nM)	95% CI (nM)	R²	Bmax ± 95% CI (%)
**WT**	0.136	±0.097	0.901	84.16 ± 12.05
**TGA123gac***	N.A.	-	0.005	2.188 ± 1.361
**TCA567ctc**	2.344	±0.843	0.984	94.19 ± 7.21
**T1g**	5.466	±1.879	0.984	52.68 ± 4.43
**G2a**	4.003	±1.245	0.989	73.92 ± 5.15
**A3g**	4.964	±2.061	0.977	58.96 ± 5.87
**C4a**	0.348	±0.187	0.955	72.26 ± 7.51
**T5c**	0.608	±0.219	0.983	71.34 ± 4.83
**C6t**	0.154	±0.09	0.943	78.51 ± 8.4
**A7c**	0.226	±0.134	0.935	83.05 ± 9.38

D: MAFG-MAFG-ARE interaction

Acceptor	Kd (nM)	95% CI (nM)	R²	Bmax ± 95% CI (%)
**WT**	1.962	±0.543	0.991	95.84 ± 5.36
**TGA123gac**	2.061	±0.62	0.989	40.41 ± 2.54
**TCA567ctc**	23.13	±3.32	0.998	67.23 ± 2.71
**T1g**	1.536	±0.455	0.990	67.34 ± 4.01
**G2a**	2.558	±0.698	0.992	93.55 ± 5.34
**A3g**	2.251	±0.556	0.993	65.17 ± 3.28
**C4a**	0.95	±0.243	0.992	71.35 ± 3.48
**T5c**	7.817	±1.75	0.995	57.24 ± 3.04
**C6t**	3.636	±0.821	0.994	69.34 ± 3.38
**A7c**	2.742	±0.854	0.988	57.49 ± 3.9
**G8c**	9.879	±4.287	0.977	68.05 ± 7.7
**C9a**	4.441	±2.07	0.973	67.8 ± 7.47
**3'-7 bp tcttaga**	4.631	±1.745	0.983	86.16 ± 7.48
**5'-7 bp cagtgtc**	2.044	±0.832	0.980	87.78 ± 7.58
**3'-5'-7 bp flip**	4.569	±1.743	0.983	86.39 ± 7.61

Equilibrium ARE-engagement curves were fit by nonlinear regression to a single-site binding function. Values are best-fit apparent K_d and Bmax estimates, and the reported ± values provide compact summaries of fitting uncertainty; exact 95% profile-likelihood limits and complete fit outputs are provided in Supporting Information Excel Sheet 1. N.A. indicates that a parameter

could not be reliably constrained. R^2 values from attempted fits are retained only to document overall fit quality.

Table S4. Comparison of kinetic fitting approaches for NRF–sMAF–ARE association traces.

A: WT ARE binding curve fitting

	Bi-exponential association fit						Single-exponential association fit			
**ARE Conc.**	$k_{obs,\ fast}$	$k_{obs,\ Slow}$	**Ratio of rate**	R^2	Y_0	**Plateau**	K_{obs}	R^2	Y_0	**Plateau**
**WT 10.0 pM**	0.04642± N.A.	0.0078± N.A.	5.952	0.9629	1.216 ± 0.051	4.268± N.A.	0.01686 ± 0.00106	0.9623	1.265 ± 0.024	3.525 ± 0.064
**WT 31.6 pM**	0.09866 ± 0.01752	0.02509 ± 0.00454	3.932	0.9943	1.166 ± 0.075	7.906 ± 0.096	0.0394 ± 0.00062	0.9918	1.569 ± 0.042	7.548 ± 0.023
**WT 100 pM**	0.1548 ± 0.0072	0.03669 ± 0.00186	4.218	0.9982	1.191 ± 0.142	22.43 ± 0.06	0.0687 ± 0.00102	0.9901	3.606 ± 0.16	21.76 ± 0.03
**WT 316 pM**	0.2379 ± 0.0057	0.03648 ± 0.00139	6.52	0.9981	2.891 ± 0.32	46.77 ± 0.07	0.103 ± 0.0026	0.9723	11.45 ± 0.57	45.33 ± 0.09
**WT 1.0 nM**	0.6055 ± 0.0251	0.01133 ± 0.00091	53.46	0.9902	41.87 ± 1.18	84.8 ± 0.6	0.03668 ± 0.00304	0.8772	65.89 ± 0.44	79.48 ± 0.26
**WT 3.16 nM**	0.4512 ± 0.0379	0.007583 ± 0.000696	59.5	0.9927	69.65 ± 0.76	101.8 ± 1.2	0.01621 ± 0.00103	0.9682	78.17 ± 0.16	94.29 ± 0.47

B: ARE mutation binding curve fitting

	Bi-exponential association fit						Single-exponential association fit			
**ARE Mutation**	$k_{obs,\ fast}$	$k_{obs,\ Slow}$	**Ratio of rate**	R^2	Y_0	**Plateau**	K_{obs}	R^2	Y_0	**Plateau**
**WT 1.0 nM**	0.6055 ± 0.0251	0.01133 ± 0.00091	53.46	0.9902	41.87 ± 1.18	84.8 ± 0.6	0.03668 ± 0.00304	0.8772	65.89 ± 0.44	79.48 ± 0.26
**TGA123gac**	0.1581 ± 0.12671	0.0001884± N.A.	839.3	0.4878	0.1985 ± 0.0948	15.48± N.A.	0.002723± N.A.	0.4848	0.2399 ± 0.0191	1.509± N.A.
**T1g**	0.8417 ± 0.0625	0.01326 ± 0.00253	63.48	0.9318	5.988 ± 0.755	15.44 ± 0.15	0.05403 ± 0.00697	0.7411	12.77 ± 0.13	14.8 ± 0.05
**G2a**	0.5021 ± 0.0258	0.01841 ± 0.00384	27.27	0.9537	0.8567 ± 0.281	7.921 ± 0.072	0.2592 ± 0.0186	0.8559	2.827 ± 0.276	7.528 ± 0.015
**A3g**	0.4265 ± 0.0172	0.01919 ± 0.00201	22.23	0.9813	1.078 ± 0.2638	11.5 ± 0.09	0.1262 ± 0.0103	0.8704	5.146 ± 0.299	10.74 ± 0.03
**C4a**	0.5057 ± 0.0119	0.01742 ± 0.00176	29.03	0.9904	3.732 ± 0.518	32.96 ± 0.16	0.2622 ± 0.0163	0.8894	11.92 ± 0.98	31.26 ± 0.05
**T5c**	0.2376 ± 0.0088	0.05085 ± 0.00832	4.673	0.9952	2.457 ± 0.246	23.71 ± 0.04	0.178 ± 0.0026	0.9887	4.164 ± 0.216	23.49 ± 0.03
**C6t**	0.3023 ± 0.0078	0.049 ± 0.00254	6.169	0.9971	6.936 ± 0.516	60.16 ± 0.06	0.1635 ± 0.004	0.9742	16.46 ± 0.76	59.21 ± 0.07
**A7c**	0.2776 ± 0.0067	0.04836 ± 0.00158	5.74	0.9982	5.015 ± 0.429	60.59 ± 0.05	0.1237 ± 0.0029	0.9754	16.04 ± 0.72	59.46 ± 0.09
**TCA567ctc**	0.1306 ± 0.0068	0.02599 ± 0.00148	5.025	0.9983	0.9251 ± 0.1099	18.87 ± 0.1	0.04901 ± 0.00078	0.9906	2.849 ± 0.118	17.82 ± 0.05
**Zipper exchange**	0.5134 ± 0.0097	0.01313 ± 0.00072	39.11	0.9962	21.44 ± 1	100.7 ± 0.6	0.083 ± 0.0085	0.8437	62.27 ± 1.81	92.29 ± 0.31

Association traces were evaluated using single- and bi-exponential fits, with model selection based on goodness of fit and parameter identifiability. Values are best-fit estimates; N.A. indicates that a 95% confidence interval could not be reliably bounded although a finite best-fit value was obtained. Exact profile-likelihood confidence limits and complete fit outputs are provided in Supporting Information Excel Sheets 3 and 4. R^2 documents overall fit quality and does not establish parameter identifiability.

Table S5. Apparent kinetic parameters for ARE variants in NRF2–MAFG–ARE binding.

Method 1

ARE	K_d (nM)	$k_{on}(nM^{-1}s^{-1})$	$k_{off}(s^{-1})$
WT	0.0762 ± 0.008	0.5342 ± 0.0972	0.0407 ± 0.00856

Method 2

ARE	K_d (nM)	$k_{on}(nM^{-1}s^{-1})$	$k_{off}(s^{-1})$
WT	0.0762 ± 0.008	0.563 ± 0.024	0.0429 ± 0.00456
T1g	2.97 ± 0.383	0.212 ± 0.026	0.630 ± 0.053
G2a	4.65 ± 1.406	0.0889 ± 0.023	0.413 ± 0.031
A3g	1.98 ± 0.534	0.143 ± 0.026	0.283 ± 0.028
C4a	0.299 ± 0.089	0.389 ± 0.028	0.116 ± 0.027
T5c	1.27 ± 0.38	0.105 ± 0.018	0.133 ± 0.018
C6t	0.261 ± 0.096	0.240 ± 0.019	0.0626 ± 0.018
A7c	0.316 ± 0.118	0.212 ± 0.019	0.0667 ± 0.019
TCA567ctc	5.22 ± 1.573	0.0210 ± 0.0054	0.110 ± 0.008
Zipper exchange	0.0762 ± 0.008	0.477 ± 0.0097	0.0364 ± 0.0036

Apparent K_d values were obtained from equilibrium fits. Apparent k_{on} was estimated either from the concentration dependence of kobs,fast (Method 1) or from fixed-concentration $k_{obs,fast}$ and independently measured K_d (Method 2); apparent k_{off} was derived from K_d and k_{on}. Reported uncertainties are approximate propagated 95% limits based on fitting uncertainty in kobs,fast and K_d. Exact source confidence limits and fit outputs are provided in Supporting Information Excel Sheets 1 and 3. N.A. indicates that a parameter could not be reliably estimated.

Table S6. Key Resources Table

Category	Resource	Source	Identifier
**Bacterial Strains**	NEB® Stable Competent E. coli	New England Biolabs	C3040
**Bacterial Strains**	Shuffle® T7 Express lysY Competent E. coli	New England Biolabs	C3030J
**Recombinant DNA & Expression System**	CIDAR Modular Cloning (MoClo) Parts Kit	Addgene	Kit #1000000059
**Recombinant DNA & Expression System**	T7-driven MoClo expression backbone	IDT	Gene fragments
**Recombinant DNA & Expression System**	Codon-Optimized gene fragments	IDT	Gene fragments
**Recombinant DNA & Expression System**	Q5® High-Fidelity DNA Polymerase	New England Biolabs	M0491
**Recombinant DNA & Expression System**	BsaI-HF®v2	New England Biolabs	R3733
**Recombinant DNA & Expression System**	BsbI-HF®	New England Biolabs	R3539
**Recombinant DNA & Expression System**	T4 DNA Ligase	New England Biolabs	M0202
**Peptides**	ALFA peptide (N-terminal Lys) KGPSRLEEEELR	GenScript	Custom synthesis
**Peptides**	ALFA peptide (C-terminal Lys) SRLEEEELRRRL	GenScript	Custom synthesis
**Fluorophores**	sulfo-Cyanine3 NHS ester	Lumiprobe	11320
**Fluorophores**	sulfo-Cyanine5 NHS ester	Lumiprobe	13320
**Fluorophores**	sulfo-Cyanine5.5 NHS ester	Lumiprobe	17320
**Fluorophores**	AF 488 NHS ester	Lumiprobe	11820
**Fluorophores**	AF 568 NHS ester	Lumiprobe	14820
**Fluorophores**	ATTO 430LS NHS ester	ATTO-TEC	AD-430LS-31
**Fluorophores**	ATTO 490LS NHS ester	ATTO-TEC	AD-490LS-31
**Fluorophores**	ATTO 590 NHS ester	Sigma-Aldrich	79636
**Chemicals & Buffers**	IPTG	Goldbio	I2481C
**Chemicals & Buffers**	Imidazole, Recrystallized	Goldbio	I-902-25
**Chemicals & Buffers**	PMSF	Goldbio	P-470-100
**Chemicals & Buffers**	Carbenicillin (Disodium)	Goldbio	C-103-5
**Chemicals & Buffers**	Kanamycin Monosulfate	Goldbio	K-120-5
**Chemicals & Buffers**	Protease Inhibitor Cocktail	Sigma-Aldrich	P8849
**Chemicals & Buffers**	Furimazine	Chem Shuttle	PBI 3939
**Chemicals & Buffers**	TCEP HCl	Goldbio	TCEP25
**Chemicals & Buffers**	DTT	Goldbio	DTT10
**Chemicals & Buffers**	Tris Base	Goldbio	T-400-500
**Chemicals & Buffers**	Ni-NTA Agarose	Qiagen	30210
**Chemicals & Buffers**	Glycerol	Sigma-Aldrich	G5516
**Plates & Consumables**	White-F-96-Well Plates	Fisher Scientific	13-882-205
**Plates & Consumables**	White-F-96-Well Plates	Fisher Scientific	14-386-022
**Plates & Consumables**	Clear-F-96-Well Plates	Fisher Scientific	13-882-202
**Software & Algorithms**	R	R Foundation	https://www.r-project.org

Table S7. Sequences and naming conventions of all ARE variants.

Labeled oligo	Sequence	Length (bp)
ARE-F-ATTO590	/5ATTO590N/CGCGTCCGCGGCCGCGTGGC	20

Forward ARE	Sequence	Length (bp)
ARE-F	AGCCTTCCAAATCCGCAGTCACAGTGACTCAGCAGAATCT	40
ARE2-F	ATCCGCAGTCACAGTGACTCAGCAGAATCTGAGCCTAGGT	40
ARE3-F	GGTGCCCTAGGCTCAGATTCTGCTGAGTCACTGTGACTGT	40
ARE4-F	CTCAGATTCTGCTGAGTCACTGTGACTGCGGATTTGGAAT	40
ARE5-F	CAGCCTTCCAAATCCGCAGTCACAGTGACTCAGCAGAATC	40
ARE-F-T1g	AGCCTTCCAAATCCGCAGTCACAGgGACTCAGCAGAATCT	40
ARE-F-G2a	AGCCTTCCAAATCCGCAGTCACAGTaACTCAGCAGAATCT	40
ARE-F-A3g	AGCCTTCCAAATCCGCAGTCACAGTGgCTCAGCAGAATCT	40
ARE-F-C4a	AGCCTTCCAAATCCGCAGTCACAGTGAaTCAGCAGAATCT	40
ARE-F-T5c	AGCCTTCCAAATCCGCAGTCACAGTGACcCAGCAGAATCT	40
ARE-F-C6t	AGCCTTCCAAATCCGCAGTCACAGTGACTtAGCAGAATCT	40
ARE-F-A7c	AGCCTTCCAAATCCGCAGTCACAGTGACTCcGCAGAATCT	40
ARE-F-G8c	AGCCTTCCAAATCCGCAGTCACAGTGACTCAcCAGAATCT	40
ARE-F-C9a	AGCCTTCCAAATCCGCAGTCACAGTGACTCAGaAGAATCT	40
ARE-F-3'-7 bp tcttaga	AGCCTTCCAAATCCGCAGTCACAGTGACTCAGCtcttaga	40
ARE-F-5'-7 bp cagtgtc	AGCCTTCCAAATCCGCAcagtgtcTGACTCAGCAGAATCT	40
ARE-F-3'-5'-7 bp flip	AGCCTTCCAAATCCGCAagattctTGACTCAGCctgttac	40
ARE-F-TGA123gag	AGCCTTCCAAATCCGCAGTCACAGgagCTCAGCAGAATCT	40
ARE-F-TCA567ctc	AGCCTTCCAAATCCGCAGTCACAGTGACctcGCAGAATCT	40
ARE-F-T1gSA7c	AGCCTTCCAAATCCGCAGTCACAGgGACTCcGCAGAATCT	40
ARE-F-G2aSC7t	AGCCTTCCAAATCCGCAGTCACAGTaACTtAGCAGAATCT	40
ARE-F-A3gST5c	AGCCTTCCAAATCCGCAGTCACAGTGgCcCAGCAGAATCT	40

Reverse Link	Sequence	Length (bp)
ARE-R	GCCACGCGGCCGCGGACGCGAGATTCTGCTGAGTCACTGTGACTGCGGATTTGGAAGGCT	60
ARE2-R	GCCACGCGGCCGCGGACGCGCCCTAGGCTCAGATTCTGCTGAGTCACTGTGACTGCGGAT	60
ARE3-R	GCCACGCGGCCGCGGACGCGGCAGTCACAGTGACTCAGCAGAATCTGAGCCTAGGGCACC	60
ARE4-R	GCCACGCGGCCGCGGACGCGCTTCCAAATCCGCAGTCACAGTGACTCAGCAGAATCTGAG	60
ARE5-R	GCCACGCGGCCGCGGACGCGGATTCTGCTGAGTCACTGTGACTGCGGATTTGGAAGGCTG	60
ARE-R-T1g	GCCACGCGGCCGCGGACGCGAGATTCTGCTGAGTCcCTGTGACTGCGGATTTGGAAGGCT	60
ARE-R-G2a	GCCACGCGGCCGCGGACGCGAGATTCTGCTGAGTtACTGTGACTGCGGATTTGGAAGGCT	60
ARE-R-A3g	GCCACGCGGCCGCGGACGCGAGATTCTGCTGAGcCACTGTGACTGCGGATTTGGAAGGCT	60
ARE-R-C4a	GCCACGCGGCCGCGGACGCGAGATTCTGCTGAtTCACTGTGACTGCGGATTTGGAAGGCT	60
ARE-R-C4t	GCCACGCGGCCGCGGACGCGAGATTCTGCTGAaTCACTGTGACTGCGGATTTGGAAGGCT	60
ARE-R-C4g	GCCACGCGGCCGCGGACGCGAGATTCTGCTGAcTCACTGTGACTGCGGATTTGGAAGGCT	60
ARE-R-T5c	GCCACGCGGCCGCGGACGCGAGATTCTGCTGgGTCACTGTGACTGCGGATTTGGAAGGCT	60
ARE-R-C6t	GCCACGCGGCCGCGGACGCGAGATTCTGCTaAGTCACTGTGACTGCGGATTTGGAAGGCT	60
ARE-R-A7c	GCCACGCGGCCGCGGACGCGAGATTCTGCgGAGTCACTGTGACTGCGGATTTGGAAGGCT	60
ARE-R-G8c	GCCACGCGGCCGCGGACGCGAGATTCTGgTGAGTCACTGTGACTGCGGATTTGGAAGGCT	60
ARE-R-C9a	GCCACGCGGCCGCGGACGCGAGATTCTtCTGAGTCACTGTGACTGCGGATTTGGAAGGCT	60
ARE-R-3'-7 bp tcttaga	GCCACGCGGCCGCGGACGCGtctaagaGCTGAGTCACTGTGACTGCGGATTTGGAAGGCT	60
ARE-R-5'-7 bp cagtgtc	GCCACGCGGCCGCGGACGCGAGATTCTGCTGAGTCAgacactgTGCGGATTTGGAAGGCT	60
ARE-R-TGA123gag	GCCACGCGGCCGCGGACGCGAGATTCTGCTGAGctcCTGTGACTGCGGATTTGGAAGGCT	60
ARE-R-TCA567ctc	GCCACGCGGCCGCGGACGCGAGATTCTGCgagGTCACTGTGACTGCGGATTTGGAAGGCT	60
ARE-R-T1gSA7c	GCCACGCGGCCGCGGACGCGAGATTCTGCgGAGTCcCTGTGACTGCGGATTTGGAAGGCT	60
ARE-R-G2aSC7t	GCCACGCGGCCGCGGACGCGAGATTCTGCTaAGTtACTGTGACTGCGGATTTGGAAGGCT	60
ARE-R-A3gST5c	GCCACGCGGCCGCGGACGCGAGATTCTGCTGgGcCACTGTGACTGCGGATTTGGAAGGCT	60
ARE-R-Blank	GCCACGCGGCCGCGGACGCG	20

WT NQO1 ARE and all mutated derivatives, with mutation codes indicating wild-type base (capital), position (number), and substituted base (lowercase). Mutations include singles, symmetric pairs, triple left/right, and 5′/3′ flank substitutions.

Additional Supporting Information Excel file

Contents:

Sheet 1 ARE binding K_d fitting

Sheet 2 Leucine Zipper K_d fitting

Sheet 3 ARE kinetic fitting

Sheet 4 Leucine Zipper Kinetic fitting

Sheet 5 Plasmid information

Citations

1. F. Sievers *et al.*, Fast, scalable generation of high-quality protein multiple sequence alignments using Clustal Omega. *Mol Syst Biol* **7**, 539 (2011).
2. J. Abramson *et al.*, Accurate structure prediction of biomolecular interactions with AlphaFold 3. *Nature* **630**, 493–500 (2024).
